# Association between dietary environmental pressures and major chronic diseases: assessment from the prospective NutriNet-Santé cohort

**DOI:** 10.1016/j.lanepe.2025.101481

**Published:** 2025-10-07

**Authors:** Emmanuelle Kesse-Guyot, Aurélien Chayre, Elie Perraud, Sylvaine Berger, Annabelle Richard, Justine Berlivet, Mathilde Touvier, Benjamin Allès, Serge Hercberg, Denis Lairon, Philippe Pointereau, Hélène Fouillet, Julia Baudry, Christian Couturier, François Mariotti

**Affiliations:** a Université Sorbonne Paris Nord and Université Paris Cité, INSERM, INRAE, CNAM, Centre of Research in Epidemiology and StatisticS (CRESS), Nutritional Epidemiology Research Team (EREN), Bobigny, France; b Solagro, Voie du Toec, Toulouse, France; c UMR PNCA, AgroParisTech, INRAE, Université Paris-Saclay, Palaiseau, France; d Inserm, INRAE, Aix Marseille Université, CV2N, Marseille, France

**Keywords:** Chronic diseases, Diet, Environmental indicators, Climate, Epidemiology, Cohort

## Abstract

**Background:**

Plant-based diets offer co-benefits for human health and the environment, but assessments often consider only specific aspects. This study comprehensively examines the links between diet-related environmental pressures and risk of chronic diseases as well as mortality.

**Methods:**

Data from a population study of 34,077 participants to the NutriNet-Santé French cohort were used. Dietary data were collected using a food frequency questionnaire, distinguishing between organic and conventional foods, and were merged with food production environmental indicators. The associations between greenhouse gas emissions (GHGe), energy demand, land occupation (LO), ecological infrastructures (EI), water use, and pesticide treatment frequency and a synthetic environmental pressures index (EPI) and incidence of cancer, cardiovascular diseases (overall, coronary and cerebrovascular diseases), type 2 diabetes and mortality were estimated using weighted multivariable cox proportional risk model.

**Findings:**

Over a mean median follow-up of 8.39 years (IQR = 5.62, 256,891 person-year), the diet's overall environmental pressures (EPI) was positively associated with the risk of all tested chronic diseases except stroke. The HR for 1 SD increment ranging from 1.15 (95% CI = 1.03–1.28) for cancer (all locations) to 1.50 (95% CI = 1.29–1.73) for coronary heart disease and type 2 diabetes, but no association with stroke or death was detected.

**Interpretation:**

Diets with low overall environmental pressures are associated with important health benefits, suggesting that food systems with lower environmental impacts could be key drivers of both environmental and health sustainability.

**Funding:**

Data were collected in the context of the BioNutriNet and TRANSFood projects supported by the 10.13039/501100001665French National Research Agency (ANR-13-ALID-0001 and ANR-21-CE21-0011-01).

**Research in context**

**Evidence before this study**

A recent systematic review has gathered existing evidence on sustainable diets, indicating that certain dietary patterns may benefit both human health and the environment. The review included studies published in English until December 2024 that examined the associations between environmental indicators and the risk of cardiovascular diseases, cancer, diabetes, and mortality. These studies were identified through a PubMed search using the terms (diet-related OR from diet OR dietary) AND (greenhouse gases OR GHG OR greenhouse gas emissions) AND (mortality OR cancer OR diabetes OR death OR cardiovascular OR chronic) AND prospective. Three studies modelled environmental indicators as exposure to health risk in prospective studies. The first, conducted in EPIC-NL, examined the prospective link between diet-related greenhouse gas emissions (GHGe) and land use and mortality. The second study, involving the entire European EPIC cohort, looked at the association between GHGe and land occupation and risk of mortality (overall and cause-specific). The third study, conducted in EPIC-Spain, examined the relationship between diet-related GHGe and the risk of cancer, cardiovascular diseases, and type 2 diabetes.

**Added value of this study**

This study is, to our knowledge, the first to explore the links between a wide range of environmental indicators distinguishing more or less sustainable diets and the risk of morbidity and mortality based on prospective cohort data with a median follow-up of 8.39 years (interquartile range = 5.62). Using data from a large (N = 34,077) population study from the NutriNet-Santé French cohort who completed a food frequency questionnaire distinguishing organic and conventional foods, we computed a synthetic environmental pressures index (EPI) of the specific environmental pressures associated with the production of diets, based on the following standardised indicators: GHGe, cumulative energy demand, LO, ecological infrastructures (EI), water use and frequency of pesticide use. The risk of death, type 2 diabetes, cardiovascular diseases (CVD) and cancer, based on validated multi-source data, was estimated for different levels of environmental pressures associated with individual diet. We found that diets with high environmental pressures, less adherent to the French dietary guidelines and EAT-Lancet diet, were positively associated with the risk of chronic diseases except for stroke that was not associated. Findings were robust in sensitivity analyses, particularly in causal inference models that simulate intervention changes in EPI.

**Implications of all the available evidence**

Our research indicates that a diet with lower environmental pressures is linked to a reduced risk of type 2 diabetes, CVD, and cancer. The co-benefits of a diet that is less detrimental to the environment vary depending on environmental indicators, as evidenced by inverse associations with water use and ecological infrastructure. However, the overall trend supports the hypothesis that such a diet also benefits human health. Emphasising these health co-benefits may appeal to individuals less concerned about environmental issues.

## Introduction

Diet plays a significant role in the burden of disease,^1^^,^^2^ with 2.1 billion people suffering from overweight or obesity.^3^ In 2021, dietary risk factors were the second leading cause of attributable deaths among women (3.48 million deaths, uncertainty intervals: 2.78–4.37) and the third among men (4.47 million deaths, 3.65–5.45) globally, highlighting regional disparities.^2^

The main dietary contributors to these attributable deaths are insufficient consumption of whole grains, fruits and vegetables, excessive intake of red and processed meats, and high sodium intake. These dietary patterns are closely linked to the onset of cancer, cardiovascular diseases, and diabetes.^1^

At the same time, activities within agri-food systems, particularly at the food production stage, significantly impact land use and the environment,^4^^,^^5^ and contribute to exceeding several planetary boundaries.^6^^,^^7^

Livestock production, including beef and dairy as well as, to a lesser extent, monogastric breeding, shows the most significant environmental impacts across many indicators: acidification, eutrophication, greenhouse gas emissions (GHGe), soil and water use, etc.^8^^,^^9^ Observational studies have shown that, in a population, diets rich or exclusively based on plant products have GHGe and land use levels well below those of meat consumers.^10^^,^^11^ Other types of scenario modelling studies confirm that diets including low quantities of or no food products of animal origin present lower environmental pressures than those of meat eaters, particularly GHGe.^10^^,^^12^^,^^13^

The recent scientific literature, based on cohort data, has documented that healthy dietary patterns may offer co-benefits for both environmental and human health.^14^, ^15^, ^16^, ^17^

These findings generally support the EAT-Lancet Commission's guidelines, which recommend a high intake of plant-based foods, including wholegrain cereals, vegetables, fruits, pulses, nuts, and seeds. They also advise reducing the consumption of animal products such as red meat, dairy, eggs, and fish, while limiting processed foods and added sugars.^5^ Research shows that following the EAT-Lancet guidelines while respecting planetary boundaries could worldwide prevent up to 11 million premature deaths annually, accounting for about 19%.^5^ In this context, many studies have assessed the links between environmental pressures or health indicators and adherence to the EAT-Lancet diet.^18^ It is also worth noting that numerous adherence indicators have been developed, and they display different properties.^19^

However, some authors emphasised that most of the evaluated co-benefits centre on air pollution and that public health researchers, epidemiologists, and health economists should aim to collaborate more actively to advance research into health co-benefits.

Furthermore, Reganold et al. conducted a literature review examining the performance of organic farming.^20^ They concluded that, although average yields are lower, organic farming significantly reduces environmental impact and offers social and ecological benefits. For instance, it is widely recognised that organic production requires less energy than conventional systems.^20^ Concerning GHGe, the disparities between organic and conventional systems are less clear and depend on the products. In addition, because organic farming yields are lower, land use tends to be higher. Diets mainly based on organic food have also been linked to a reduced risk of some chronic diseases.^21^

In this context, we examined the relationship between food-related environmental pressures and various indicators, including greenhouse gas emissions (GHGe), land occupation (LO), energy use, pesticide application, water use, and ecological infrastructures—considering the production method (organic and conventional). Additionally, we employed a composite indicator designed to reflect the overall environmental impact and potential disparities among different indicators and health risks across a broad cohort, utilising both observational and counterfactual methodologies. Importantly, the individual environmental indicators were estimated by considering the farming method of the food.

## Methods

### Study population

This study was conducted on a sample of adults from the web-based prospective NutriNet-Santé cohort, which aims to investigate the complex relationships between dietary habits and health and disease.22 Participants are volunteers aged over 15 years recruited from the general French population. In the present study, data collected between 2014 and 2024 were used.

### Ethical approval

The study is registered at https://clinicaltrials.gov/ct2/show/NCT03335644, conducted according to the Declaration of Helsinki guidelines and approved by the Institutional Review Board of the French Institute for Health and Medical Research (IRB-Inserm) and by the French National Commission for Information Technology and Liberties (Commission Nationale de l’Informatique et des Libertés) (CNIL n°908450/n°909216). Each participant provides an electronic informed consent form in the NutriNet-Santé cohort before enrolment.

### Data collection

Data on age, sex, highest educational attainment, occupation, income per household unit per month, marital status, smoking habits, and physical activity were collected at cohort enrolment and annually thereafter using validated questionnaires.^22^^,^^23^ Physical activity was measured using the International Physical Activity Questionnaire (IPAQ).^24^ Tobacco consumption, expressed in pack-years, was also calculated. Validated anthropometric questionnaires provided information on height and weight.^23^ Family history, including the history of cancer, stroke, myocardial infarction, and type 2 diabetes (T2D) among parents and siblings, was collected.

Dietary data (baseline point) were collected between June and December 2014 using a 264-item self-administered semi-quantitative food frequency questionnaire (Org-FFQ). This enables the specification of whether the food was organic (as defined by the official European standards and label) or conventionally produced.^25^ This dietary measurement tool is based on a previously validated FFQ,^26^ improved by a five-point scale to assess the proportion of organic food consumption in the diet.^25^ For each food item, participants reported the frequency with which it was consumed as organic by selecting one of the following options: “never”, “rarely”, “half-of-the-time”, “often” or “always” in response to the question ‘*How often was the product of organic origin*?’. Each modality was assigned a weight, i.e., 0, 25, 50, 75, and 100%, respectively. Nutrient and total energy intakes were calculated using a published food composition table.^27^ To identify underreporting or overreporting participants, we estimated basal metabolic rate by Schofield equations according to sex, age, weight, and height collected at enrolment in the study.^28^ Energy requirement, accounting for physical activity level and basic metabolic rate, was compared with energy intake. The ratio of energy intake to energy requirement was calculated, and individuals with ratios below or above cut-offs (0.35 and 1.93) were excluded.^25^

Two dietary scores, sPNNS-GS2, reflecting the adherence to the French dietary guidelines^29^ and the Planetary Health Dietary Index (PHDI)^30^ were computed. Details are provided in Supplemental Method 1.

All covariates were collected as close in time to the completion of the FFQ.

Environmental pressures were estimated by combining food consumption (except for drinking water) with six indicators: GHG emissions, LO, cumulative energy demand (CED), ecological infrastructures (EI) reflecting biodiversity, pesticide use (using treatment frequency index (TFI)) and water use (related to irrigation). Life cycle assessments from the DIALECTE database were used to calculate food-related GHGe, CED, and LO. The computation procedures for these three indicators have been extensively described elsewhere.^31^ Various databases (French annual agricultural statistics, FAOSTAT,^32^ Surveys on farming practices in France, Agribalyse,^33^ Graphic parcel register, BD Haie, BD Forêt®, effectives wetlands, Agreste^34^) were used to calculate EI (Ecological Infrastructures constitute a network of elements incorporated into the agricultural environment to harmonise production with biodiversity preservation), pesticide use, and water use for both organic and conventional food. Economic and biophysical allocations, along with cooking and edibility coefficients, were applied to agricultural raw materials. Details and references are provided in Supplemental Method 2.

For each indicator, a higher value reflects greater pressure, except for EI. Based on the 6 environmental normalised indicators (reversed for EI), a summarised environmental pressures index (EPI) was computed, with a higher value reflecting greater pressure. The procedure is explained in Supplemental Method 3.

Health events were identified using a multisource approach. Participants were asked to report significant health events by completing a yearly health questionnaire, a specific biannual questionnaire, or using a specific interface on the study website at any time. After reporting a major health event such as cardiovascular diseases or cancer, participants were asked to provide all medical records and anatomopathological reports to confirm the diagnosis. If necessary, the study physicians contacted the participants' general practitioners or relevant medical institutions to collect further information and to validate the reported cases. In addition, the data collected within the NutriNet-Santé study were linked to medico-administrative databases of the Caisse Nationale de l’Assurance Maladie (social health insurance system), thereby limiting potential bias for participants who may not report their disease to the study investigators. Finally, additional and exhaustive information on mortality (date and cause of death) was obtained from the countrywide Centre d'épidémiologie sur les causes médicales de Décès (CépiDc) database. All cases were defined as the first occurrence of cancer (except basal cell carcinoma, not considered as cancer), cardiovascular diseases (CVD), considering all CVD, stroke and coronary heart diseases (CHD) specifically, T2D and death, occurring between the completion of FFQ and August 2024.

Details are provided in Supplemental Method 4.

### Statistical analysis

To be included in the present study, participants had to have completed the Org-FFQ and reside in France to ensure their eligibility for the French census weighting process. To conduct a disease-specific analysis, the prevalent cases (type 1 and 2 diabetes in the case of the T2D analysis) of the respective disease were excluded. The participants’ flowchart is shown in Supplemental Figure S1.

For each sex, a weighting was determined by on the 2009 national census considering age, occupational categories, area of residence and whether or not the household included at least one child (<18 y), marital status, and educational attainment, using the iterative proportional fitting procedure, to adjust the percentage of individuals in each stratum to the actual percentage in the French population. Weights were calculated using the “CALage sur MARges” procedure (SAS CALMAR macro).^35^

To illustrate the profiles of the participants in the cohort, compared with the French population, the weights according to characteristics are presented in Supplemental Table S1. Then all analyses are weighted.

For descriptive purposes, the mean (SD) or percentage of baseline characteristics - including sociodemographic, lifestyle, and environmental and dietary indicators - are presented for the overall weighted sample and the weighted quintile of EPI. Tests for differences were calculated using the Mantel-Haenszel χ^2^ test for dichotomous or ordinal variables, or linear contrasts from ANOVA for numeric variables.

In addition, dietary consumptions (standardised to 2000 kcal) are presented per weighted quintile of EPI.

Cox proportional hazard models, with age as the primary time scale, were used to evaluate the association between each environmental indicator or the EPI and the incidence of cancer, CVD, T2D, and all causes of mortality (except suicides, fatal accidents, and unknown causes). Participants contributed person-time from the Org-FFQ completion until the date of the studied health event, the date at which the last questionnaire was completed, the date of death, or August 2024, whichever occurred first. Hazard ratios (HR) and 95% confidence intervals (CI) were computed for each model. Exposure variables were environmental indicators considered as continuous variables (per one SD) and weighted sex-specific quintiles. Cox proportional hazard assumption was verified using the rescaled Schoenfeld-type residual method,^36^ as shown in Supplemental Figure S2. The log-linearity and dose–response of the relationships between environmental indicators and hazard ratios for chronic diseases were appraised using restricted cubic splines,^37^ as shown in Supplemental Figure S3. The selection of confounding factors is based on the literature of the major determinants of dietary behaviours and the health events studied.

In the main analyses (model M1), models were adjusted for age (time-scale), sex (male/female), physical activity level (low, moderate, high), smoking status (current smoker, former smoker, non-smoker), cumulative number of pack-years of cigarette smoking, energy intake (continuous, kcal/d), educational attainment (< High school diploma, High school, ≤3 years after high school, >3 years after high school), living status (cohabiting or not), occupational status (retired, unemployed, farmer/merchant/craftworker/company director, manual worker, employee/manual worker, intermediate profession, managerial staff/intellectual profession), monthly household income per consumption unit (non-disclosed, <1200 €, 1200–1800 €, 1800–3700 €, ≥3700 €), body mass index (BMI) (continuous, kg/m^2^), and family history of cancer, diabetes or cardiovascular diseases depending on the analysis. For the cancer analysis, height (continuous, cm) and, for women, the number of children, hormone replacement, age at menarche and contraceptive use at enrolment were included in the model.

We derived marginal survival curves, which can be interpreted as the counterfactual survival function that would have been observed if the entire population had been exposed to food with high environmental pressures. Finally, marginal structural models (MSM) were constructed to estimate the “causal” effect of environmental pressures on several health events while considering confounding factors.^38^^,^^39^ The MSM approach mimics the design of a randomised controlled trial (RCT) by creating pseudo-randomisation through statistical reweighting, thereby reducing confounding bias that would otherwise preclude causal inference in observational data. In short, the MSM approach involved three key stages: the estimation of propensity scores, i.e. the inverse probability of treatment weights (IPTW), using logistic regression models that included the major covariates. The weights are composed of two propensity scores, which estimate either the probability of ‘receiving’ an exposure as a function of the covariates or the probability of censoring. More details on the method and assumptions are provided in Supplemental Material 5.

Several sensitivity analyses are described in Supplemental Method 5. SAS 9.4 (SAS Institute) and R® version 4.0.4 (R 197 Foundation) were used for the analyses; tests were two-sided and considered statistically significant when the *P*-value was <0.05.

### Role of funding source

The funders had no role in study design, data collection and analysis, manuscript preparation, or the decision to submit for publication.

## Results

### Characteristics of the sample and diets

The weighted mean of baseline age of the study population (n = 34,077) was 48.4 years (SD = 16.3). After weighted adjustment, women composed approximately 52% of the sample.

The characteristics of the EPI by weighted sex-specific quintile and in the overall sample are shown in Table 1. The EPI was positively associated with age and negatively associated with educational attainment. Executive or higher intellectual professions had lower EPI, while retired people had higher EPI. The Environmental Pressures Index was also inversely associated with income level. The environmental and dietary characteristics by weighted sex-specific quintile and in the overall sample are shown in Table 2. The weighted mean of the EPI for 2000 Kcal is 13.90/100 (SD = 3.77) (Table 2).

**Table 1:** Baseline sociodemographic and lifestyle data across weighted quintiles of Environmental Pressures Index (NutriNet-Santé cohort, 2014, n = 34,077).^a^

	All	Q1	Q2	Q3	Q4	Q5
N (weighted)						
Cut-off						
Men		<10.42	10.42-<13.12	13.12-<15.79	15.79-<20.49	≥20.49
Women		<9.07	9.07-<11.47	11.47–13.99	13.99-<17.89	≥17.89
EPI, median [IQR]						
Men	14.44 (98.44)	21.33 (70.12)	8.24 (8.41)	11.67 (2.69)	14.28 (2.67)	17.67 (4.69)
Women	12.18 (85.21)	7.45 (9.07)	10.27 (2.4)	12.61 (2.52)	15.71 (3.91)	21.33 (70.12)
Sex, %Women	52.30	52.39	52.98	51.77	52.09	52.29
Age (y), mean (SD)	48.39 (16.23)	45.91 (16.52)	47.12 (16.66)	47.78 (16.12)	50.46 (15.11)	50.66 (16.28)
**Education**, (%)						
<High school diploma	59.63	50.18	58.84	61.74	58.46	68.88
High school	15.51	19.45	15.09	13.30	15.78	13.93
≤3 years after high school	11.85	13.65	11.72	12.29	13.26	13.65
>3 years after high school	13.01	16.73	14.35	12.66	12.50	8.86
**Occupation**, (%)						
Retired	27.48	22.82	26.31	27.21	31.43	29.62
Executive or higher intellectual profession	9.11	11.65	9.44	7.88	9.61	6.96
Craftsman, trader, business manager, farmer	4.46	6.95	4.87	3.59	3.94	2.97
Intermediate occupation	14.49	17.40	16.02	12.68	14.62	11.78
Employee/manual worker	31.14	27.76	29.92	34.56	28.77	34.64
Unemployed	4.25	4.36	5.76	5.25	2.55	3.34
Never Unemployed	9.07	9.06	7.69	8.83	9.09	10.68
**Monthly income per household unit, (%)**						
<1200€	14.16	13.73	16.84	15.23	10.57	14.47
1200–1800€	28.64	26.24	28.05	30.26	31.21	27.43
1800–3700€	24.23	25.56	25.60	19.86	26.52	23.64
>3700€	14.98	15.56	13.75	15.41	16.27	13.90
Missing data	17.98	18.90	15.76	19.23	15.43	20.57
Marital status, % cohabiting	80.22	77.53	79.05	78.82	82.12	83.55
**Tobacco use, %**						
Never-smokers	47.37	54.11	50.08	47.75	42.25	42.73
Former smokers	39.95	32.27	40.19	36.53	46.19	44.57
Current smokers	12.68	13.62	9.73	15.72	11.57	12.70
**Physical activity, %**						
High	33.99	31.87	32.32	34.19	36.10	35.44
Moderate	30.59	31.96	31.76	30.88	31.94	26.45
Low	21.10	24.43	21.98	21.23	18.90	18.97
Missing data	14.32	11.74	13.95	13.70	13.06	19.14
BMI (kg/m^2^)	24.94 (5.91)	23.75 (4.56)	24.85 (8.66)	24.78 (4.93)	25.14 (4.63)	26.16 (5.65)

Abbreviations: BMI, body mass index; EPI, environmental pressures index; IQR, interquartile range.

All P-value <0.001 except for sex.

a Value are weighted means (SD) or % as appropriate, except otherwise is specified.

**Table 2:** Environmental and dietary indicators across weighted quintiles of Environmental Pressures Index (NutriNet-Santé cohort, 2014, n = 34,077).^a^

	All	Q1	Q2	Q3	Q4	Q5
EPI Cut-off						
Men		<10.42	10.42–<13.12	13.12–<15.79	15.79–<20.49	≥20.49
Women		<9.07	9.07–<11.47	11.47–13.99	13.99–<17.89	≥17.89
EPI standardised to 2000 kcal	13.90 (3.77)	10.64 (2.86)	12.88 (2.82)	14.16 (3.47)	14.93 (2.67)	16.85 (3.91)
***Individual environmental indicators***						
GHGe (kgCO_2_eq/d)	4.44 (2.79)	2.04 (0.84)	3.14 (1.17)	4.07 (1.37)	5.06 (1.44)	7.88 (3.87)
Energy demand (MJ/d)	18.59 (8.49)	9.71 (2.50)	13.73 (2.39)	17.31 (2.67)	21.16 (3.11)	30.98 (8.88)
Land occupation (m^2^/d)	11.49 (7.47)	5.51 (2.24)	8.23 (3.14)	10.61 (4.08)	13.01 (4.20)	20.05 (11.11)
Pesticides use (FTI/d)	23.09 (12.35)	11.51 (5.22)	17.02 (6.42)	20.87 (5.87)	26.80 (6.98)	39.16 (13.62)
Water use (m^3^/d)	0.25 (0.12)	0.16 (0.06)	0.21 (0.09)	0.24 (0.08)	0.28 (0.08)	0.37 (0.15)
Ecological infrastructures (m^2^/d)	0.80 (0.57)	0.38 (0.20)	0.56 (0.26)	0.74 (0.36)	0.90 (0.36)	1.40 (0.87)
***Dietary indicators***						
Energy Intake (kcal/d)	2112.22 (709.09)	1492.13 (400.56)	1775.82 (399.44)	2002.45 (472.80)	2314.00 (429.01)	2971.39 (746.08)
Alcohol (g/d)	8.01 (12.63)	5.92 (10.15)	7.06 (10.62)	6.86 (12.35)	9.84 (13.01)	10.33 (16.12)
% of organic food in the diet	0.26 (0.27)	0.36 (0.32)	0.29 (0.30)	0.24 (0.24)	0.22 (0.22)	0.17 (0.21)
sPNNS-GS2	2.03 (3.70)	4.23 (2.62)	3.39 (3.06)	2.51 (3.35)	0.99 (3.17)	−0.96 (3.84)
PHDI	90.78 (13.16)	94.38 (14.53)	91.67 (14.41)	90.40 (12.79)	89.37 (10.94)	88.08 (11.86)
Total proteins (g/d)	97.20 (39.83)	60.36 (16.38)	77.04 (16.91)	92.28 (20.37)	107.36 (21.39)	148.65 (47.45)
Animal proteins (g/d)	67.05 (37.18)	33.50 (16.16)	49.14 (18.48)	63.43 (19.12)	76.88 (21.05)	112.02 (46.91)

Abbreviations: EPI, summarized Environmental Pressures Index; FTI, frequency treatment index; GHGe, greenhouse gas emissions; PHDI, planetary health dietary index; sPNNS-GS2, simplified *Programme National Nutrition-Santé* Guidelines-score 2. All P-values for linear contrast across quintiles are <0.05. Data are weighted for the Census.

a Values are unadjusted weighted mean (SD) except otherwise is specified.

By construction, EPI was positively associated with each of its constituent contributors from pressure indicators. Higher ecological infrastructure was observed despite the inversion of the indicator in the Environmental Pressures Index computation (Supplemental Method 5).

The diet of the participants in the 5th weighted quintile of EPI (compared to the 1st) exhibited +286% higher food-related GHGe, +219% higher CED, +264% higher LO, +272% higher EI, +240% higher pesticide use and +129% water use (Table 2).

Participants in the 5th weighted quintile of EPI (compared to the 1st) had higher energy intake (+99%) and lower nutritional quality of the diet, they also had higher consumption of total and animal protein intakes (Table 2).

**Fig. 1:**
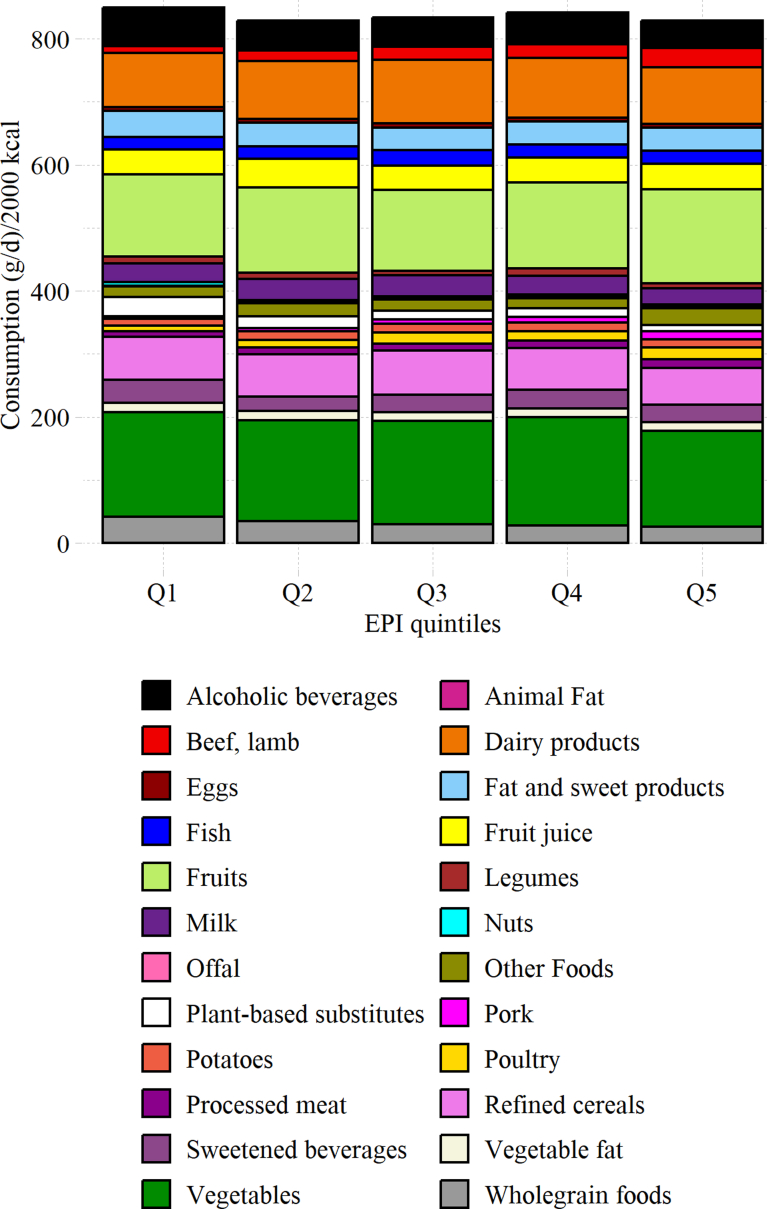
**Food consumption (g/d) per 2000 kcal across weighted quintile of summarized Environmental Pressures Index (NutriNet-Santé study FFQ, 2014, n = 34,077)**. Values are per 2000 kcal/d weighted on the French National Census.

The average food consumptions per 2000 kcal across EPI quintiles are presented in Fig. 1, and crude values are presented in Supplemental Table S2. When considering consumption per 2000 kcal, diets with a high level of EPI were characterised by high consumption of meat (pork, ruminants, poultry, offal and processed meat). Conversely, the consumption of wholegrain foods and pulses was significantly lower in diets with the highest EPI than in diets with the lowest one.

### Environmental pressure and health risk

**Fig. 2:**
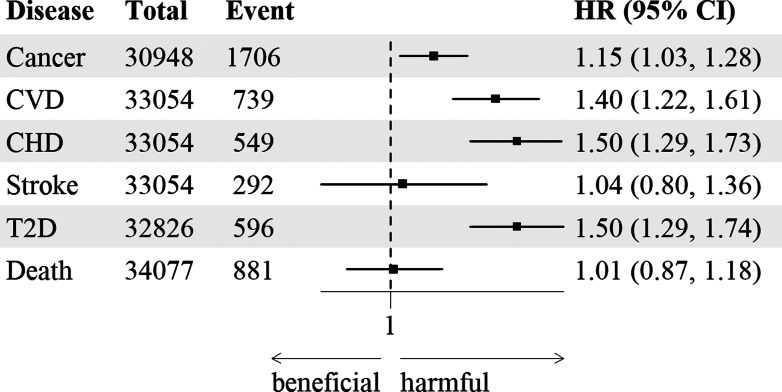
**Prospective Association between the summarized Environmental Pressure Index and risk of chronic diseases and mortality (NutriNet-Santé study, 2014–2024)**. Abbreviations: CHD, Coronary heart disease; Cardiovascular diseases, CVD; EPI, Environmental pressures Index; T2D, type 2 diabetes. The stroke and coronary heart disease sub-analyses also included non-validated events, which explains why the sum is greater than the CVD total, which includes validated events only. Values are number (total and disease cases), HR (95% CI). HR (95% CI) are extracted from a multivariable Cox proportional hazards model weighted on national Census and adjusted for age (time-scale), sex (male/female), physical activity level (low, moderate, high), smoking status (status as smoker, former smoker and non-smoker, and number of pack-year), number of 24-h dietary records (continuous), educational attainment (<high-school degree, ≤3 years of higher education, >3 years of higher education), living status (cohabiting or not), occupational status (retired, unemployed, farmer/merchant/craftworker/company director, employee/manual worker, intermediate profession, managerial staff/intellectual profession, never employed), monthly income per unit consumption of the household (non-communicated, <1200 €, 1200–1800 €, 1800–3700 €, ≥3700 €), energy intake (continuous, in kcal/d), body mass index (BMI) (continuous, in kg./m^2^), and family history of cancer, diabetes or cardiovascular diseases depending on the analysis. For the cancer analysis, height (continuous, in m) and, for women, number of children, hormone replacement and contraceptive use were included in the model.

The weighted median (IQR) of follow-up times were 8.04 (5.74), 8.15 (5.72), 8.21 (5.71) and 8.39 (5.62) for cancer (n cases = 1706), CVD (n cases = 739), T2D (n cases = 596) and death (n cases = 881), analyses, respectively. The associations between EPI and health risk are presented in Fig. 2 and Table 3. A higher value of the Environmental Pressures Index was positively associated with the risk of chronic diseases, i.e. cancer, CVD (all), CHD and T2D, but no association was detected for stroke and death (see Fig. 2 and Table 3). The HR for 1 SD ranged from 1.15 (95% CI = 1.03–1.28) for the risk of cancer (all locations) to 1.50 (95% IC = 1.29–1.73) for the risk of coronary heart disease and 1.50 (95% IC = 1.29–1.74) for the risk of T2D.

**Table 3:** Association between Environmental Pressures Index and risk of chronic diseases and death, main analyses (NutriNet-Santé cohort, France**,** 2014–2024 n = 34,077).^a^

	Continuous variable^b^	P-value	Sex-specific		Quintile			P-trend^c^
			Q1	Q2	Q3	Q4	Q5	
Cancer								
n cases (unweighted)	1706		282	301	379	411	333	
Person-year	226,017		41,939	44,040	42,964	44,192	44,547	
Model 1 (main)	1.15 (1.03–1.28)	0.01		0.71 (0.58–0.87)	1.46 (1.22–1.75)	0.97 (0.79–1.19)	1.21 (0.95–1.55)	0.02
Cardiovascular diseases								
n cases (unweighted)	739		115	136	156	180	152	
Person-year	244,734		43,708	45,442	45,313	46,507	46,666	
Model 1 (main)	1.40 (1.22–1.61)	<0.0001		1.29 (0.99–1.68)	1.38 (1.05–1.81)	1.67 (1.26–2.20)	1.94 (1.38–2.72)	0.0001
Coronary heart diseases								
n cases (unweighted)	549		86	103	106	133	121	
Person-year	244,734		43,708	45,442	45,313	46,507	46,666	
Model 1 (main)	1.50 (1.29–1.73)	<0.0001		1.25 (0.94–1.66)	0.94 (0.69–1.29)	1.55 (1.15–2.10)	2.19 (1.53–3.14)	0.0001
Stroke								
n cases (unweighted)	292		54	53	65	64	56	
Person-year	244,734		43,708	45,442	45,313	46,507	46,666	
Model 1 (main)	1.04 (0.80–1.36)	0.76		1.75 (1.08–2.82)	2.34 (1.45–3.77)	1.37 (0.80–2.34)	1.26 (0.65–2.41)	0.76
Type 2 diabetes								
n cases (unweighted)	596	71	90	125	141	169	596	
Person-year	244,248		42,709	46,162	44,326	46,054	46,554	
Model 1 (main)	1.50 (1.29–1.74)	<0.0001		0.37 (0.26–0.52)	0.95 (0.71–1.28)	1.13 (0.85–1.52)	1.41 (0.99–2.02)	0.0001
Death								
n cases (unweighted)	881	146	160	187	190	198	881	
Person-year	256,891		45,850	48,355	47,737	49,366	49,337	
Model 1 (main)	1.01 (0.87–1.18)	0.85		0.95 (0.74–1.21)	1.20 (0.94–1.54)	0.93 (0.71–1.21)	0.95 (0.68–1.33)	0.79

a The main model (M1) is a weighted multivariable Cox proportional hazard model adjusted for age (time-scale), sex (male/female), physical activity level (low, moderate, high), smoking status (status as current smoker, former smoker and non-smokers, and number of pack-year), energy intake (continuous, in kcal/d), number of 24-h dietary records (continuous), educational attainment (<high-school degree, ≤3 years of higher education, >3 years of higher education), living status (cohabiting or not), occupational status (retired, unemployed, farmer/merchant/craftworker/company director, employee/manual worker, intermediate profession, managerial staff/intellectual profession, never employed), monthly income per unit consumption of the household (non-communicated, <1200 €, 1200–1800 €, 1800–3700 €, ≥3700 €), body mass index (BMI) (continuous, in kg/m^2^), and family history of cancer, diabetes or cardiovascular diseases depending on the analysis. For the cancer analysis, height (continuous, in m) and, for women, number of children, hormone replacement and contraceptive use were included in the model. HR (Hazard Ratio) and 95% CI (95% confidence interval) are derived from multivariable Cox proportional hazard, Q: Quintile.

b By increment of 1SD.

c P-value of Wald test for quintile as an ordinal variable.

Results of the sensitivity analyses for the EPI are shown in Supplemental Table S3. Most findings yielded results similar (magnitude of the hazard ratio and statistical significance) to the main model (M1), notably those without energy adjustment and early cases exclusion (sensitivity analyses 1 and 2, respectively), except that the association with cancer risk was attenuated. Findings were also similar to the main findings in the models with capping weight (sensitivity analyses 3).

In models employing a marginal structural model (sensitivity analysis 4), which simulate a randomised trial, and in models without weighting (sensitivity analysis 5) for Census data, the findings were similar to the main models but achieved statistical significance only for T2D risk.

The adjusted survival curves for a fixed covariate profile are presented for each health event in Supplemental Figure S4. The differential risk across EPI quintiles is quite distinct for the risk of diabetes, CVD, CHD and mortality, especially from age 65 onwards. For the risk of cancer and stroke, the confidence intervals are wide.

The associations for each environmental indicator are presented in Supplemental Figure S5. GHGe, CED, LO, and pesticide use were all positively associated with cancer, CVD (in particular CHD), and T2D risks, while GHGe was additionally inversely associated with stroke. The last two indicators, Water use and EI, exhibited completely different profiles. Water use was found to have a negative association with cancer risk. In addition, EI, which indicates biodiversity levels, where higher values are preferable, was positively linked to risks for cancer, CVD, CHD, and T2D, with no association observed regarding mortality.

## Discussion

This study employed data from a large adult cohort to assess the relationship between various environmental pressures related to dietary production and associated morbidity and mortality. While most previous studies focused on GHGe and LO, this research examined multiple environmental indicators and distinguished between organic and conventional production methods. A higher diet-related Environmental Pressures Index (EPI) was associated with increased risks of cancer, CVD, and T2D. Additionally, the marginal survival curves and marginal structural models, which simulate a randomised trial, assessed how changes in dietary EPI exposure impact health risks among similar individuals, reinforcing findings from the traditional approach.

The dietary profiles of participants with a lower EPI closely aligned with the recommendations of the EAT-Lancet Commission,^5^ characterised by low meat intake (including poultry and red meat), moderate dairy intake and high consumption of fruits, vegetables and whole grains. However, processed meat consumption was relatively high, and pulses consumption was relatively low, likely influenced by Westernised eating patterns.

Studies quantifying the co-benefits of dietary changes for human and planetary health mainly rely on modelling approaches that estimate averted deaths associated with more sustainable diets through simulation or identify healthier and more sustainable diets using optimisation.^12^^,^^17^^,^^40^, ^41^, ^42^ For instance, Springmann et al. conducted a modelling analysis on delayed deaths resulting from changes in food consumption and their subsequent environmental pressures.^40^ Additionally, a review by Wilson et al. listed the optimisation studies used to identify healthy and sustainable diets and described those aimed at distinguishing them. However, all these studies help identify the best dietary profiles and their potential benefits, but do not assess observable effects in real-world settings.^12^

Furthermore, our findings are consistent with the scientific literature, which connects less environmentally impactful diets with improved health outcomes. Diets that follow the EAT-Lancet recommendations, i.e., within planetary boundaries, have been associated with a lower risk of diabetes, CVD, stroke, cancer, and death.^18^^,^^43^, ^44^, ^45^, ^46^, ^47^, ^48^, ^49^, ^50^, ^51^, ^52^, ^53^, ^54^ Caution is advised when interpreting our stroke findings, as limited statistical power due to a low number of cases affects this outcome. However, a study investigating the link between an adherence index for the EAT-Lancet diet and stroke observed similar results, indicating a trend towards increased stroke risk with greater adherence to the diet.^43^

In fact, limited research has measured co-benefits using individual-level data to comprehensively outline the underlying related diets.^14^^,^^55^ A previous study with a large sample from the European EPIC cohort, followed for 14 years, revealed that diet-related GHGe and LO were positively associated with overall and cause-specific mortality, notably by cancer and CVD.^56^ Another study in Spain reported higher risks of cancer, CHD and T2D among participants with higher diet-related GHGe but did not investigate stroke risk.^57^ Our data generally align with these studies.

Our study presents an added value, by highlighting additional key factors not previously considered in the literature. While agriculture uses about 70% of global water withdrawals and is a major driver of biodiversity loss and degradation,^8^ biodiversity conservation and water resource use have received insufficient attention within the co-benefits approach for human and planetary health.

Here, we found that, unlike most other environmental footprints, water resource preservation conflicted with health, as diets higher in water demand were associated with a lower cancer risk. This is probably because water use mainly results from fruit consumption,^5^^,^^58^^,^^59^ which is protective against cancer of the upper aerodigestive tract and allows high fibre intake associated with reduced risk of colorectal cancer.^60^ This finding aligns with previous research showing that environmental co-benefits are not ubiquitous in relation to water use and sustainable diets.^14^^,^^61^^,^^62^ Likewise, the preservation of biodiversity, measured by a proxy such as the ecological infrastructures (where higher values are preferable), is considered more crucial in meat-rich diets; however, connecting it to land use (as highlighted in our summary indicator) is essential.

Interestingly, our findings suggest that the frequency of pesticide treatment is positively linked with the risk of cancer, cardiovascular diseases (CVD), and T2D. To the best of our knowledge, this particular indicator, which mostly reflects the pressure on the environment from pesticide use and, at least partly, participants' exposure, has not been extensively studied. However, it can be somewhat interpreted in light of existing research that shows a connection between exposure to pesticide residues and the risk of non-communicable diseases.^63^^,^^64^ It should be noted that the TFI measures a very different aspect from exposure to pesticide residues through food. For example, in our data, TFI values of animal products are high due to pesticides used in feed, yet pesticide residues in these products tend to be low.^65^ Conversely, for plant-based foods rich in pesticide residues, the TFI indicates dietary exposure. While the associations between pesticide pollution or biodiversity loss and health outcomes have not been thoroughly explored, a recent review compiling scientific knowledge on soil and water pollution related to CVD risk concluded that deforestation, excessive fertiliser use, plastics, and pesticides, alongside their environmental release, lead to soil and water contamination pollution.^66^ These factors significantly contribute to biodiversity loss, reduce ecosystem sustainability and food crop yields, and jeopardise human health.^67^

Public health serves as a crucial leverage point to promote the adoption of sustainable lifestyles, particularly by emphasising the links between dietary choices, environmental impact, and individual health.^68^^,^^69^ In fact, framing the climate debate from the perspective of human health proves to be a strong motivator for personal engagement, especially in high-income countries^68^ or among demographic segments that might remain passive when faced with climate-only arguments.^70^ Furthermore, delivering messages from a health-focused perspective elicits more positive emotional responses and gains greater support than discussions that focus solely on environmental or climate threats.^71^ A public health communication strategy that clearly emphasises the health benefits of sustainable lifestyles enhances both individual and collective motivation, thus supporting the shift towards more environmentally sustainable eating habits.^68^ This approach generates momentum that encourages commitment and long-term behavioural change. Health professionals and policymakers can play a key role by leading targeted initiatives to facilitate this vital transformation.^70^

This study presents several limitations. First, the study sample consisted of volunteers with particular traits, notably a predominance of women and educated individuals and is not representative of the general population. Similarly, the dietary patterns within the NutriNet-Santé cohort are often healthier than those observed in representative French national surveys. While a diverse range of dietary profiles can be captured with this large sample, census data weighting was employed to address this concern. Second, the sample size was quite limited, which restricted the statistical power for examining cancer sites broadly, and the number of strokes was low compared to other health outcomes. Another limitation is that the environmental indicators were evaluated solely at the production level; however, it is known that most pressures occur during this phase.^8^ Then, as with any observational study, residual confounding bias may still exist despite attempts to account for various confounding variables; therefore, caution is necessary when interpreting the results. More critically, the MSM assumes that this type of bias is absent, which is a key requirement considered. Also, caution must be exercised when interpreting the results, as the decisions taken when allocating indicator values (as mentioned in the Supplementary Material) can directly have a significant impact on the results, and residual confounding may have occurred. Finally, it is possible that risk alpha was inflated with multiple comparisons. However, our analyses were hypothesis-driven, and the number of analyses for each exposure-outcome pair was limited.

Furthermore, the large sample size, long follow-up period, and detailed characterisation of the sample enabled high-quality analyses. It is also noteworthy that using causal inference models, such as survival marginal models, produced robust results. Lastly, regarding environmental pressures, the matching of consumption data with environmental indicators considered whether foods were produced through conventional or organic farming methods, allowing for accurate estimates. In addition to common factors like GHGe and LO, we also explored associations with ecological infrastructure and pesticide use.

### Conclusion

In our study, using a composite index of six environmental indicators that accounted for two farming methods, we found that diets with higher environmental pressures were linked to increased risks of cancer, cardiovascular diseases, and type 2 diabetes. These findings emphasise that while certain environmental necessities, such as water resources and biodiversity preservation, may conflict with reducing some health risks, the overall relationship between environmental footprint and morbidity supports a win–win scenario, i.e. strong alignment of benefits. The health benefit could be an additional lever to promote more environmentally friendly practices. Promoting a shift towards sustainable diets for human health could also help engage segments of the population that are less responsive to environmental concerns.

## Contributors

EK-G designed the study, AC, SB, AR, and CC developed the database related to environmental indicators. EK-G, MT, and SH designed and conducted the NutriNet-Santé study; EK-G conducted the statistical analyses and wrote the manuscript. All authors provided critical comments on the manuscript. EK-G, JBau, JBer, MT, AC, SB, AR, and CC have an access to the raw data and verified the data. EK-G takes the responsibility for integrity of the data and the accuracy of the data analysis, she is the guarantor. She had primary responsibility for the final content, and all authors read and approved the final manuscript.

## Data sharing statement

Researchers from public institutions can submit a request to have access to the data for strict reproducibility analysis (systematically accepted) or for a new collaboration, including information on the institution and a brief description of the project to collaboration@etude-nutrinet-sante.fr. All requests will be reviewed by the steering committee of the NutriNet-Santé study. If the collaboration is accepted, a data access agreement will be necessary and appropriate authorisations from the competent administrative authorities may be needed. In accordance with existing regulations, no personal data will be accessible. R/SAS code is available without restrictions upon request at collaboration@etude-nutrinet-sante.fr.

## Declaration of interests

No conflict of interest is declared for any of the authors.

## Appendix A Supplementary data

Translated abstract
