# Supplementary material for: Association between dietary environmental pressures and major chronic diseases: assessment from the prospective NutriNet-Santé cohort
Source: Lancet Reg Health Eur. 2025 Oct 7;59:101481. doi: 10.1016/j.lanepe.2025.101481 (PMC12538917; doi:10.1016/j.lanepe.2025.101481)
Supplement: Translated abstract [file mmc1.docx]

**Editor disclaimer:** *This translation in French was submitted by the authors and we reproduce it as supplied. It has not been peer reviewed. Our editorial processes have only been applied to the original abstract in English, which should serve as reference for this manuscript.*

Les régimes alimentaires riches en végétaux offrent des co-bénéfices pour la santé humaine et l’environnement, mais les évaluations considèrent souvent uniquement quelques indicateurs environnementaux, en particulier les émissions de gas à effet de serre. Cette étude les liens entre les pressions environnementales liées à l’alimentation et la morbi-mortalité.

Des données provenant d’une étude de population de 34 077 participants de la cohorte française NutriNet-Santé ont été utilisées. Les données alimentaires ont été recueillies à l’aide d’un questionnaire de fréquence alimentaire distinguant les aliments biologiques et conventionnels, puis fusionnées avec des indicateurs environnementaux de la production alimentaire. Les associations entre les émissions de gaz à effet de serre (GES), la demande énergétique, l’occupation des sols (LO), les infrastructures écologiques (IE), l’utilisation de l’eau, la fréquence de traitement par pesticides, et un indicateur synthétique de pression environnementale (IPE), ainsi que l’incidence de cancers, de maladies cardiovasculaires (globales, coronariennes et cérébrovasculaires), le diabète de type 2 et la mortalité, ont été estimées à l’aide d’un modèle de risques de Cox multivariable pondéré.

Sur une période de suivi médiane de 8,39 ans (IQR=5,62, 256 891 années-personnes), les pressions environnementales globales du régime alimentaire (IPE) était positivement associée au risque de toutes les maladies chroniques testées, sauf les accidents vasculaires cérébraux. Le hazard ratio pour une augmentation d’un écart-type allait de 1,15 (IC à 95%=1,03-1,28) pour le cancer (toutes localisations) à 1,50 (IC à 95%=1,29-1,73) pour les maladies coronariennes et le diabète de type 2, mais aucune association avec les accidents vasculaires cérébraux ou la mortalité n’a été détectée.

Les régimes alimentaires à faible pression environnementale globale sont associés à d’importants bénéfices pour la santé, ce qui suggère que des systèmes alimentaires ayant un impact environnemental réduit pourraient être un levier clé pour la durabilité environnementale et sanitaire.
