# Supplementary material for: Association between dietary environmental pressures and major chronic diseases: assessment from the prospective NutriNet-Santé cohort
Source: Lancet Reg Health Eur. 2025 Oct 7;59:101481. doi: 10.1016/j.lanepe.2025.101481 (PMC12538917; doi:10.1016/j.lanepe.2025.101481)
Supplement: Supplementary Figures and Tables [file mmc2.docx]

**Supplemental Material**

**Kesse-Guyot et al.**

[Supplemental Method 1: Dietary indexes computation 2](#_Toc194662805)

[Supplemental Method 2: Environmental impact indicators by agricultural product relating to pesticides, water and ecological infrastructures 3](#_Toc194662806)

[Supplemental Method 3: Description of the Environmental Pressures Index 8](#_Toc194662807)

[Supplemental Method 4: Case ascertainment. 8](#_Toc194662808)

[Supplemental Method 5: Description of the sensitivity analyses 9](#_Toc194662809)

[Supplemental Figure 1: Flowchart, NutriNet-Santé cohort, France, 2014-2024 FU: Follow-up; CVD: cardiovascular diseases; T2D: Type 2 diabetes 10](#_Toc194662810)

[Supplemental Figure 2: Correlations between Schoenfeld residuals and timescale (age, y) from multivariable Cox models between EPI and risk of chronic diseases and mortality, NutriNet-Santé study, 2014-2024, (n = 34,077). 11](#_Toc194662811)

[Supplemental Figure 3: Restricted cubic spline plots of the association between EPI and risk of chronic diseases and mortality, NutriNet-Santé study, 2014–2024 (n = 34,077). 12](#_Toc194662812)

[Supplemental Figure 4: Standardized survival curves by quintiles of EPI for chronic diseases or mortality, NutriNet-Santé cohort, France, 2014-2024 13](#_Toc194662813)

[Supplemental Figure 5: Association between each individual environmental indicator and chronic diseases and mortality (NutriNet-Santé, 2014-2024) 14](#_Toc194662814)

[Supplemental Table 1: Description of weight according to characteristics categories (NutriNet-Santé cohort, France, 2014 (n = 34,077) 16](#_Toc194662815)

[Supplemental Table 2: Dietary Consumption according to EPI quintiles (NutriNet-Santé cohort, 2014, n=34,077)^1,2^ 17](#_Toc194662816)

[Supplemental Table 3. Association between EPI and risk of chronic diseases and death, main analyses (NutriNet-Santé cohort, France, 2014–2024 (n = 34,077)^1^ 18](#_Toc194662817)

[Supplemental Table 4. Association between EPI and risk of chronic diseases and death, main and sensitivity analyses (NutriNet-Santé cohort, France, 2014–2024 (n = 34,077)^1^ 20](#_Toc194662818)

[References 23](#_Toc194662819)

# Supplemental Method 1: Dietary indexes computation

**sPNNS-GS2**

In March 2017, as part as the development of the fourth *Programme National Nutrition Santé* (PNNS, 2017-2021), the *Haut Conseil de Santé Publique* (HCSP) published a report updating the 2001 PNNS recommendations ^1^ based on scientific literature about the relationships between diet and long-term health and a model created by the *Agence nationale de sécurité sanitaire de l'alimentation, de l'environnement et du travail* (Anses). ^2^ These new recommendations provide dietary guidelines, with 6 food groups to favor ("fruits and vegetables", "nuts", "legumes", "wholegrain", "milk and dairy products", and "fish and seafood") and 6 items to moderate ("meat", "processed meat", "added fats", "sugary products", "beverages", and "salt"). The nutrition experts who were involved in developing the guidelines defined the thresholds and corresponding scores. These thresholds and related scores are defined so as that following the guidelines is associated with one point, whereas not following them is scored zero points. To increase the power of discrimination, half-points are allocated in a linear fashion above the guideline thresholds. However, an exception was made for milk and dairy products, and fish. As, the relationship between these foods and health is non-linear, allocated points form a parabolic relationship. ^3^

The sPNNS-GS2 emphasizes on the distinction between bonus components (healthy foods considered beneficial, which have a positive adequacy score, e.g. legumes) and malus components (unhealthy food thought to be avoided, which have a negative moderation score, e.g. salt).

**The Planetary Health Diet Score**

The Planetary Health Diet Score^4^ (PDHI) evaluates compliance with a dietary framework established by the EAT-Lancet Commission.^5^ It comprises 15 components, with each rated on a scale of 0 to 10 points. Notably, legumes and soy-based products contribute with a weight of 0·5, bringing the maximum score to 140. Scores are assigned on a scale from 0 to 10, depending on whether the consumption aligns with the established ranges; if it does not, the score defaults to either 0 or 10, depending on the component scoring with the consumption (ascending or descending).

|  | **Target of EAT-Lancet diet reference**^1^ | | **Criteria for scoring (g/d)** | |  |
| --- | --- | --- | --- | --- | --- |
| C**omponent** | g/d (for 2500 kcal/d) | % total energy intake | **0 (min)** | **10 (max)** | **Weight in the PDHI** |
| Whole grain | 232 (0%–60%^2^) | 811 | 0 | ≥75 or ≥90 g/d^3^ | 1 |
| Tubers | 50 (0–100) | 39 | ≥200 | ≤50 | 1 |
| Vegetable | 300 (200–600) | 78 | 0 | ≥300 | 1 |
| Whole fruit | 200 (100–300) | 126 | 0 | ≥200 | 1 |
| Dairy foods | 250 (0–500) | 153 | ≥1000 | ≤250 | 1 |
| Red/processed meat^4^ | 14 (0–28) | 30 | ≥100 | ≤14 | 1 |
| Chicken and other poultry | 29 (0–58) | 62 | ≥100 | ≤29 | 1 |
| Eggs | 13 (0–25) | 19 | ≥120 | ≤13 | 1 |
| Fish and shellfish | 28 (0–100) | 40 | 0 | ≥28 | 1 |
| Nuts | 50 (0–75) | 291 | 0 | ≥50 | 1 |
| Non-soy legumes | 50 (0–100) | 172 | 0 | ≥100 | 0·5 |
| Soybean/soy foods | 25 (0–50) | 112 | 0 | ≥50 | 0·5 |
| Unsaturated added fat | 40 (20–80) | 354 (14·2%) | ≤3·5%^2^ | ≥21%^2^ | 1 |
| Saturated added fat | 11·8 (0–11·8) | 96 (3·8%) | ≥10%^2^ | 0%^2^ | 1 |
| Added sugar | 31 (0–31) | 120 (4·8%) | ≥25%^2^ | ≤5%^2^ | 1 |

Abbreviations: TEI, total energy intake

^1^ As defined by the Lancet Commissions ^5^

^2^ of total energy intake

^3^ ≥75 g/d for female, ≥90 g/d for males

^4^ Including beef, lamb, pork

# Supplemental Method 2: Environmental impact indicators by agricultural product relating to pesticides, water and ecological infrastructures

Methodological choices and assumptions:

The newly developed environmental indicators have been calculated for 84 agricultural products, including 73 plant products and 11 animal products. Indicators for fishery and aquaculture products have not been calculated.

Two farming systems were considered: "conventional" (i.e., non-organic) and "organic" agricultural methods as defined in European Commission (EU) 2018/848. ^6^

For products produced in France, French references are used, while foreign references are used for imported products. The term "organic agriculture" refers to production methods that meet the standards set out in the European Union’s regulations. ^6^ All other production methods that do not meet these standards are classified as "conventional".

We ensured that the sources correspond to the geographical areas where the food consumed in France was produced. The FAO trade matrices ^7^ are used to identify the main countries producing and exporting to the European Union and France. The supply balances were compiled using the MOSUT ^8^ tool designed by SOLAGRO, based on data from the supply balances ^9^ between 2017 and 2020. These assessments were then used to categorize products into two groups: those that are "mainly imported" (imports/resources > 50%) and those that are "mainly produced" in France (imports/resources < 50%). In total, references for 12 products produced outside France were sought: Coffee, Cocoa, Tea, Orange, Grapefruit, Lemon, Rice, Olive, Walnut, Green Bean, Soy, and Tomato.

For each product defined as “mainly imported”, the main producing countries were identified based on production and trade data available in FAOSTAT. ^10^ In the case where data on production yields, pesticide use, and water consumption (mostly irrigation) were available for only one of the main producing countries, that data were used for calculation. For example, soy flour used in animal feed is mainly imported from Brazil in conventional farming and from Togo, India, or Ukraine in organic farming. ^7^ For certain products such as rice, walnuts, and green beans, no references were available for the main producing and exporting countries to France, so French references were used by default.

In 2021, organic farming, accounting for 10·5% of French agricultural land, was included in the statistical data without distinction between conventional and organic methods. Therefore, the average production from the annual statistics was assumed to represent conventional production. Thus, the average yield in "conventional" agriculture was calculated by dividing the total quantity produced by the total cultivated area. Average organic yields were calculated using yield loss coefficients from Dialecte, ^11^ Agribalyse or scientific publications. For imported products and buckwheat, the FAO average yield was used as yield for conventional farming.

To quantify the environmental indicators for animal products, the land used to produce their feed was considered. The Agribalyse ® database 3·1 ^12^ provides information on animal food products consumed in France. It uses livestock feed data and regional yields to calculate indicators for products like milk, eggs, and meat. A biophysical allocation method was applied to allocate resources to co-products. ^13^ Although organic systems for turkey, duck, rabbit, goat's milk, and sheep's milk were not considered in this study, case studies have been adapted for these types of farms.

Computation of the indicators:

***Pesticide footprint***

The plant health treatment frequency index (TFI) is a standardized indicator that measures the frequency of pesticide use for a given crop. The TFI, derived from farmers’ reported practices, was adapted from the Danish indicator. ^14^ It is defined as the number of reference doses applied per spatial unit over a specified period. In most cases, the spatial unit is the plot, with the period being the crop year. This indicator can then be aggregated at different spatial and temporal scales. Furthermore, the index can be segmented by family or type of plant protection product, by type of treatment, or by type of crop. It can also be broken down into different segments, according to the type of product used: herbicide, insecticide, fungicide, seed treatment, biological control, or other. By aggregating substances with different modes of action, the TFI provides a comprehensive measure of overall pesticide use.

For further details on the French standardized calculation of the TFI, see to the Ministry of Agriculture and Food's methodological guide. ^15^ In this study, we assessed three TFI: total TFI (excluding biological control products), herbicide TFI and non-herbicide TFI (excluding biological control products).

Biological control products were excluded from the analysis, as our primary focus is on the environmental impact of synthetic pesticides, which generally pose a greater environmental risk compared to biological alternatives. ^16,17^

The pesticide footprint quantifies the land area treated with pesticides to produce 1 kg of a given commodity. The calculation method differs for plant and animal products:

- For crops, the footprint is obtained by multiplying the average Total TFI by the inverse of the crop yield.
- For animal products, we first determined the average area of land required to produce 1 kg of product per crop type. This area was then multiplied by the corresponding TFI and multiplied by the inverse of their yield.

The result is expressed as pesticide-impacted area equivalents, referred to as pesticide use. The area impacted by pesticide use is referred to as the pesticide footprint. This includes the herbicide footprint use and the non-herbicide use.

The data used for the computation of pesticides footprint are summarized below (Table 1).

**Table 1 Data sources for the computation of the pesticide’s footprint**

|  | **Data sources** |
| --- | --- |
| TFI herbicides, excluding herbicides et total (excluding biological control), conventional | 1. - French surveys on plant protection practices (2017 for field crops, 2018 for fruit growing and vegetables, 2019 for vine growing) 2. -Technical documents and scientific literature 3. -Agribalyse ® 4. -Surveys on the use of plant protection products in Spain |
| TFI herbicides, excluding herbicides et total (excluding biological control) organic | - French surveys on phytosanitary practices (for fruit growing, 2019 for vine growing)   1. -Technical documents from the DEPHY networks |
| Average conventional yield | 1. -Average yield between 2017 and 2021 from annual agricultural statistics (assimilated to average conventional yield) |

***Water use***

The Water indicator groups two indicators to characterize agricultural production:

- **Water requirements for crop production** (irrigation);

- **Water requirements for livestock production** (watering, and cleaning of facilities).

Several methods have been developed to assess water footprint in recent years: ^18–20^

- Pfister et al.^21^( "Withdrawal to Availability" method). It considers both **water consumed (EC)** and **water returned (ER)** to the environment, treating returned water as part of the overall water footprint.

- Hoekstra et al. (”Consumption to Availability” methods). ^20,22^ This method excludes water withdrawals and returns from the calculation, focusing only on water consumption. means that the quantities of water withdrawn and returned to the system (ER) are excluded from the calculation.

- AWaRe method ^23^ (Available Water Remaining). This method developed as part of the latest generation of water footprint assessments, calculates water consumed (EC) relative to the water available in the region studied.

Our objective was to quantify the total water withdrawn for food production, rather than just the water consumed by plants. Although some of the withdrawn water returns to the system, water withdrawal represents the volume of water temporarily unavailable for other uses, creating potential competition with other sectors. Therefore, irrigation water used was estimated using the "withdrawal to availability" calculation method developed by Pfister et.al. ^18^

Irrigation water use is significant: in France, representing nearly 3 billion m^3^ per year, including 1 billion m^3^ for maize irrigation and 306 million m^3^ for soft wheat irrigation. ^24^ The irrigation water indicator highlights the pressure on a product's water resources as a function of its production method (organic and conventional) and practice (m^3^/ha).

The indicator is calculated using the total amount of irrigation water used in mainland France for the crop under study, divided by its total production.

$$water for irrigation\left( \frac{m^{3}}{\mathrm{kg}}of product \right)= \frac{total quantity of water withdrawn}{total production per crop}$$

Total quantity of water used for irrigation is determined by multiplying the irrigated area of the crop in question by the quantity of irrigation applied per hectare:

$${total quantity of water withdrawn}_{region}={irrigated area}_{region} \times{quantity of irrigation per ha}_{region}$$

The irrigated area data are sourced from the Agricultural Census (AC) available on the Agreste website. ^25^ The most recent available data (2020) were used, as they best reflect current irrigation practices and average climatic conditions. The data were analyzed by region and by crop.

Due to lack of data, to calculate irrigation water usage, it was assumed that the percentage of irrigated area and the amount of water per hectare is the same for both organic and conventional farming. This assumption was necessary because comparative data on organic vs. conventional irrigation practices are scarce. Moreover, irrigation water management depends on various factors as: irrigation technologies (sprinklers, drippers, etc.), soil textures (sandy, loamy, etc.), organic matter percentage, soil preparation, etc. ^26^ While irrigation needs may differ between organic and conventional systems, the available data did not allow for a precise differentiation. The only factor we were able to account for was climate, using regional irrigation data. ^27^ The water use per kg of product is influenced by both yield variation and geographical distribution of production. For example:

- 39% of conventional and 34% of organic maize is cultivated in “Nouvelle Aquitaine” region where the water amount is 199 mm/ha whereas,
- 10% of conventional and 21% of organic maize is cultivated in “Pays de la Loire” region where the water amount is 111 mm/ha.

Although total water use per ha for maize in France is greater for conventional than organic, the yield difference ( ̴30% lower for organic maize) results in higher water use per kg of organic maize.

To estimate the organic irrigated areas, the total irrigated area per region has been multiplied by the proportion of organic farmland in that region.

Irrigation water data (mm/ha) are not systematically available for all crop types in all region. Then data were available from cropping surveys, ^27^ They were used directly. For the missing data, additional sources were used and validated by experts.

Water indicator for livestock farming (excluding irrigation) was calculated using data from Agribalyse 3.1®, which provides estimates of water used for watering and facility cleaning per liter of milk or kg of meat. The calculation follows the ReCiPe 2016 Midpoint (H) method: Water consumption - market for tap water. ^28^

***Ecological infrastructure (EI)***

EI refers to landscape features that support biodiversity and ecosystem services. These features can be classified into several types:

- Linear or surface tree formations (hedges, copses, trees, agroforestry, etc.),

- Grassed areas (extensive grassland, areas under environmental cover, etc.),

- Cultivated areas (environmental set-aside, extensive arable strips, etc.),

- Ruderal areas (low walls, terraces, grassed paths),

- Wetlands (ponds, springs, wet ditches).

To develop the EI indicator, we standardized all features using a common characteristic variable that could be linked to food or fodder production areas. To ensure robustness and comprehensive coverage, the following features were included:

- Surface area of hedges and linear tree elements

- Surface area of grassed strips (buffer strips along watercourses)

- Surface area of forest edges resulting from an intersection between the BD Forêt® and the GPR (Graphic parcel register)

- Surface area of copses

- Surface area of wet meadows (share of wetlands in permanent pasture by livestock production area)

- Surface of grazed woodland (share of grazed woodland in permanent pasture by livestock production area)

- Surface of fallow land (> 5 years old) (code J6S in GPR 2021)

- Surface of dry-stone walls

- Surface of ponds

Each of these EI was identified using spatial data and quantified in terms of surface area, either by characterizing the surface area directly (wet grasslands, for example) or by multiplying it by an effect coefficient applied to the linear length of the EI.

Priority was given to applying coefficients derived from the CAP11 Ecological Interest Areas. ^29^

Grassed strips and fallow lands were assigned to crops in proportion to the length of intersection with the adjacent plots.

Wet meadows and grazed woodland were only assigned to livestock production.

We did not assign any ecological infrastructure to the imported products.

**Table 2 Ecological infrastructures data source and unit**

| **Type** | **unit** | **Data source** | **Used coefficient** |
| --- | --- | --- | --- |
| Hedges | Linear meter | Intersection between the plots of the 2021 GPR (Graphic Parcel Register) and the "hedges" layer of the BD TOPO (IGN). | 1 m = 20 m² |
| Grass strips | Square meter | Plots of the 2021 GPR coded BTA | Real surface area |
| Woodland edge (excluding poplar groves) | Linear meter | Intersection between the plots of the 2021 GPR and the linearized BD FORET (IGN) layer | 1 m = 8 m² |
| Wet meadows | Square meter | Intersection between the plots of permanent pastures coded PPH, SPH, SPL, BOP, CAE, CEE in the 2021 GPR and the inventory of effective wetlands from the SIG Wetlands Network: https://sig.reseau-zones-humides.org/ | Actual surface area m^2^ inventoried as "wet" per m^2^ of permanent pastureland |
| Fallow land over 5 years old | Square meter | Plots of the 2021 GPR coded J6S | Actual area m² fallow per m² adjacent crop |
| Grazed woods | Square meter | Plots of the 2021 GPR coded BOP | Actual surface area m² of woodland grazed per m² of permanent pasture |
| Groves | Square meter | Intersection between the plots of the 2021 GPR and the "Zone de vegetation" layer of the BD TOPO (IGN), where the "nature" field is equal to "Bois" | 1 m^2^ = 1,5 m² |
| Dry-stone walls | Linear meter | Intersection between the plots of the 2021 GPR and the "Construction linéaire” layer of the BD TOPO (IGN), where the "nature_detaillee" field is equal to "Mur de pierres sèches" | 1 linear meter = 1 m² |
| Seas | Square meter | Intersection between the plots of the 2021 GPR and the "Plan d’eau" layer of the BD TOPO (IGN), where the "nature" field is equal to "Mare" | 1 m^2^ = 1·5 m² |

Abbreviations: GPR, graphic parcel register ; BD TOPO IGN, Institut national de l'information géographique et forestière topographic database

***Comparison with national figures***

To validate the results obtained, the calculated indicators per kilo of raw product were multiplied by the quantities produced in mainland France or by the quantities imported (for soy) and compared to national data.

▶In 2024, the total pesticide footprint of the plant products considered in conventional agriculture is estimated at 57.7 million hectares in France. ^30^ As part of the ADONIS project, Solagro used the same calculation method (based on TFI) to assess the pesticide use frequency at the municipal level. The sum of the ADONIS TFIs for mainland France is 60.1 million hectares. The difference can be explained by the fact that the considered products do not cover all treated crops (e.g., seed production is excluded). Additionally, the 57·7 million hectares estimate does not include organic farming, or feed production for livestock. Despite these limitations, the results align closely with national estimates, validating the order of magnitude of the calculated pesticide indicators.

▶The total annual irrigation water use for the considered products is 2.7 billion m^3^.

According to the “Banque nationale des prélèvements quantitatifs en eau”, ^24^ the volume of water withdrawn for irrigation in France was 3.1 billion m^3^/year between 2017 and 2020. Since the considered products account for 85% of irrigated land, this confirms the validity of calculated irrigation water indicator. Using the "Water consumption" indicator from the Agribalyse ReCiPe 2016 Midpoint (H) method, water use for livestock watering and cleaning buildings is estimated at 234 million m^3^.

There are few recent references on overall water use. A 2001 study by the French Institute for the Environment estimated water consumption at ~400 million m³. ^31^

However, since 2001, the number of cattle and pigs has fallen. IDELE (Institut de l’élevage, French livestock institute) now estimates that “*the water footprint of dairy and meat products is of the order of 1 to 3 liters of water per liter of milk and 30 to 50 liters of water per kilo of live meat (at the farm gate)*”, which confirms the order of magnitude used in in this project, but with a higher footprint for milk (6L for 1L of milk) and a lower footprint for meat (27L for 1kg of meat). ^32^ These estimates require further consolidation.

According to these figures, nearly 60% of the watering and washing water footprint is accounted for by dairy cattle, 15% by beef cattle and 11% by pigs.

▶To validate the EI, we applied the coefficients calculated for each EI to national agricultural production in mainland France. The expected results should correspond to the length or surface area of the EI in France (excluding areas that are not considered int the perimeter, such as seed production, sorghum, etc.).

The obtained values are higher than those of the source data, which come from the intersection between the plots in the GPR and the EI layer. This discrepancy arises because the GPR covers only 80-85% of cultivated areas in France. Therefore, EI coverage is likely higher than those intersected by the GPR. Wet grassland extrapolated from animal products covers 302,638 ha, compared to an identified total of 328,574 ha, representing 92% agreement.

Grazed woodland extrapolated from animal products covers 229,537 ha, compared to an identified total of 290,048 ha, representing 79% agreement.

# Supplemental Method 3: Description of the Environmental Pressures Index

A synthetic Indicator of Environmental Pressures (EPI) was calculated by normalizing each indicator to a scale of 0 to 1. For agroecological infrastructures, a high value is considered positive; therefore, the result was subtracted from one. These standardized values were then summed and rescaled to stay within the same range of 0 to 1. The final sum was then multiplied by 100 to produce an EPI that ranges from 0 to 100. A higher EPI indicates a greater environmental impact. The distribution of the EPI is showed below:


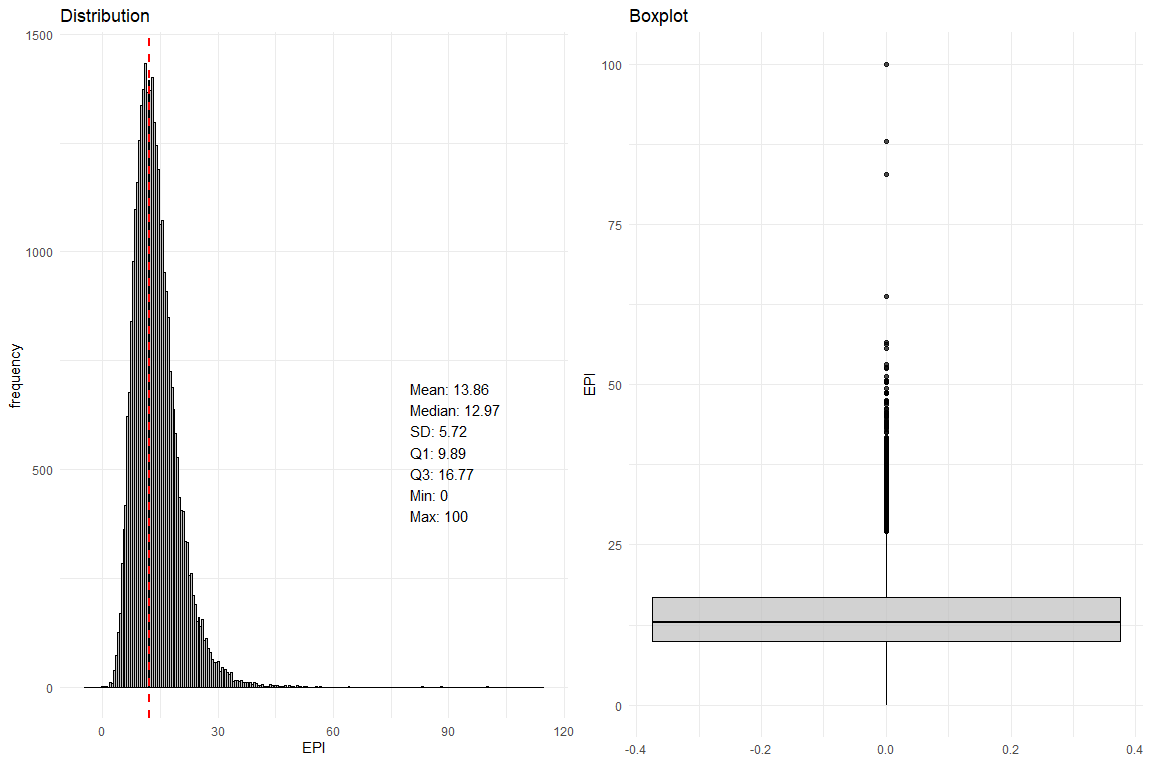


The correlation between EPI and individual environmental indicators are shown below:


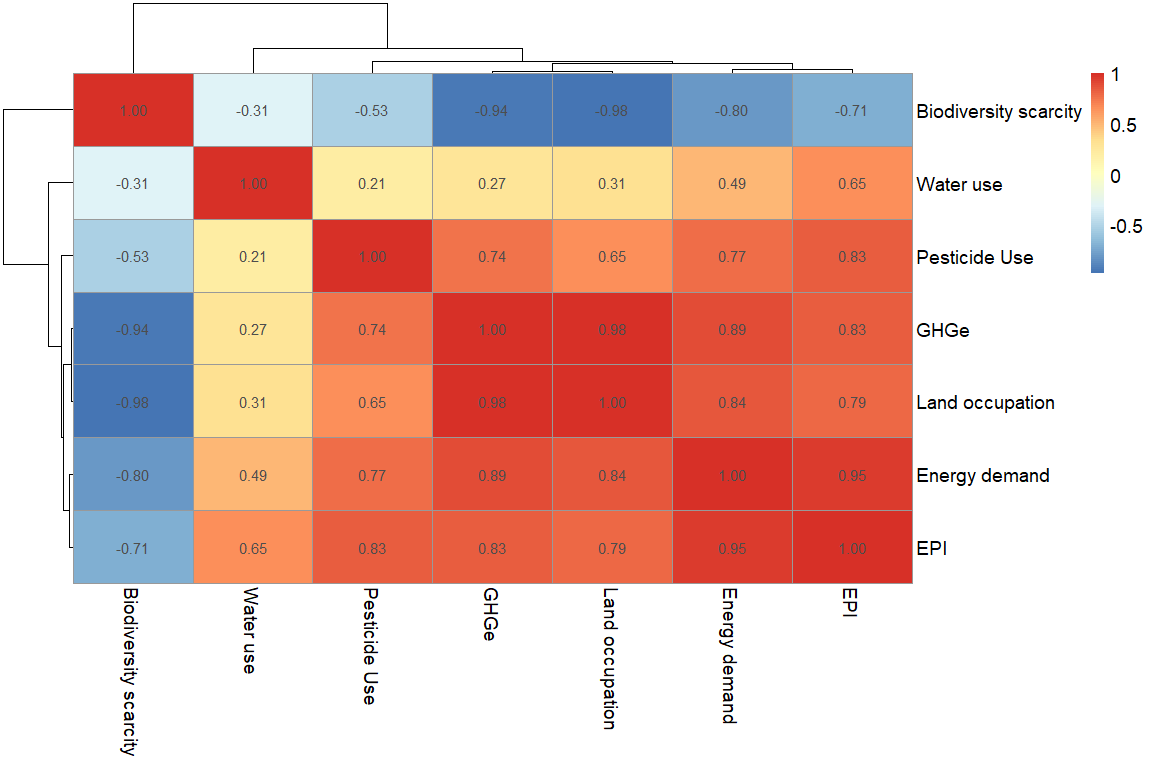
Abbreviations: EI ecological infrastructures; EPI, environmental pressures index; GHG, greenhouse gas emissions. Biodiversity scarcity is (100 - Ecological infrastructures) to facilitate reading and ensure that all indicators point in the same direction

# Supplemental Method 4: Case ascertainment.

Participants were asked to declare major health events through the yearly health questionnaire, a specific health check-up questionnaire every six months, or at any time through a specific interface on the study website. They were also asked to declare all currently taken medications and treatments via the check-up and yearly questionnaires. A search engine with an embedded exhaustive Vidal® drug database facilitates medication data entry for the participants. Besides, our research team was the first in France to obtain authorization by Decree in the Council of State (n°2013-175) to link data from our general population-based cohorts to medico-administrative databases of the National Health Insurance. Thus, data from the NutriNet-Santé cohort were linked yearly to these medico-administrative databases, providing detailed information about medication reimbursement and medical consultations.

CVD and cancer cases were classified according to the International Chronic Diseases Classification, 10^th^ Revision, Clinical Modification.

Specifically, all cancers except basal cell carcinoma were included, and the CVD included acute coronary syndrome, angina pectoris, myocardial infarction, stroke, and transient ischemic attack including only validated events. For stroke and coronary heart disease sub-analyses, non-validated events were also included explaining why the sum is greater than the CVD total, which includes validated events only.

Cases of T2D were identified using a multi-source approach, in which participants were asked to self-report their T2D status during follow-up, and to report whether they were taking any T2D medication (or reimbursement of T2D medication detected from SNIIRAM) or had hyperglycemia in the biological data along with one T2D medication use.

All T2D cases were primarily detected through the participants’ declaration of a T2D diagnosis by a physician and/or diabetes medication use in follow-up questionnaires. The questions were: “Have you been diagnosed with T2D (if yes, indicate the date of diagnosis)” and “Are you treated for T2D ?”. ATC codes considered for T2 diabetes medication were A10AB01, A10AB03, A10AB04, A10AB05, A10AB06, A10AC01, A10AC03, A10AC04, A10AD01, A10AD03, A10AD04, A10AD05, A10AE01, A10AE02, A10AE03, A10AE04, A10AE05, A10AE30, A10BA02, A10BB01, A10BB03, A10BB04, A10BB06, A10BB07, A10BB09, A10BB12, A10BD02, A10BD03, A10BD05, A10BD07, A10BD08, A10BD10, A10BD15, A10BD16, A10BF01, A10BF02, A10BG02, A10BG03, A10BH01, A10BH02, A10BH03, A10BX02, A10BX04, A10BX07, A10BX09, A10BX10, A10BX11, A10BX12.

In addition to the abovementioned questions about the diagnosis of T2 diabetes and/or a medication report, two additional sources of confirmation were considered. Initially, the connection with medico-administrative databases validated over 80% of the surveyed cases (ICD-10 codes E11). Furthermore, in the group providing blood samples during the clinical/biological examination, 85·3% of those exhibiting elevated fasting blood glucose levels (≥1·26 g/L) had reliably reported a diagnosis of T2 diabetes and/or were receiving medication. However, elevated blood glucose levels without any confirmation of a T2 diabetes diagnosis or treatment were deemed insufficiently specific to classify the participant as having T2 diabetes case.

# Supplemental Method 5: Description of the sensitivity analyses

Several sensitivity analyses for testing robustness were conducted. 1) A model (M2) similar to M1 (main model) but without adjustment for energy intake was used. 2) We used the principal model (model M1) after removing early cases occurring during the first two years of follow-up to limit potential reverse causality. 3) The data were re-analyzed after capping weight > 95^th^ percentile to this value. ^33^ 4) We also conducted marginal structural modelling (MSM) to build counterfactual models. Detailed methodology is provided below. Causal inference techniques are designed to predict the effect of a potential intervention using randomized experiments or observational data. Marginal structural models are a form of causal inference technique involving a multi-stage estimation procedure designed to control for the effect of confounding variables, particularly when the exposure distribution is unbalanced. ^34,35^ Observations are weighted by individual weights to create a pseudo-population in which exposure is no longer associated with confounding variables, thus replicating a randomized study used to estimate a causal effect.

Such models considered two weights based on inverse probability weighting implying the probability of exposure and the probability of censoring. Two weights based on inverse probability weighting implying the probability of exposure and the probability of censoring are combined as follows:

$$\mathrm{SW}^{E,C}=\mathrm{SW}^{E}\times\mathrm{SW}^{C}=\frac{f\left( E_{0} \right)}{f\left( E_{0} | A_{0} \right)}\times\frac{\Pr\left[ C=0 | E_{0} \right]}{\Pr\left[ C=0 | E_{0},A_{0} \right]}$$

The combined weights $\mathrm{SW}^{E,C}$are calculated by multiplying the stabilized inverse probability of exposure weight ($\mathrm{SW}^{E})$ and the stabilized inverse probability of censoring weight ($\mathrm{SW}^{C})$. These probabilities were obtained through linear and logistic regressions, with f(x) denoting a probability density function assuming Gaussian distribution. ^36^ The variables E, A and C were defined as follows: E represents exposure, A is a vector of covariates, and C is the indicator variable for censoring during the follow-up. Both the numerators were used for stabilization process and were derived from distinct models. The probability of exposure was estimated using the covariates of the model M1.

Untruncated weight may lead to doubtful findings. Thus, participants with a weight >10 were excluded.^33^

If we assume that there were no measurement errors during the study, no unmeasured confounders and that the models used for estimating weights are correctly specified, then the application of the combined weights to the study participants will result in the generation of a pseudo-population. This population ensures that the distribution of diet-related environmental pressures is free from any confounding factors.

Marginal Structural Model allows to provide adjusted survival curves to account for residual confounding and censoring. ^34,35^ Weight for census data and weight for MSM were then combined.


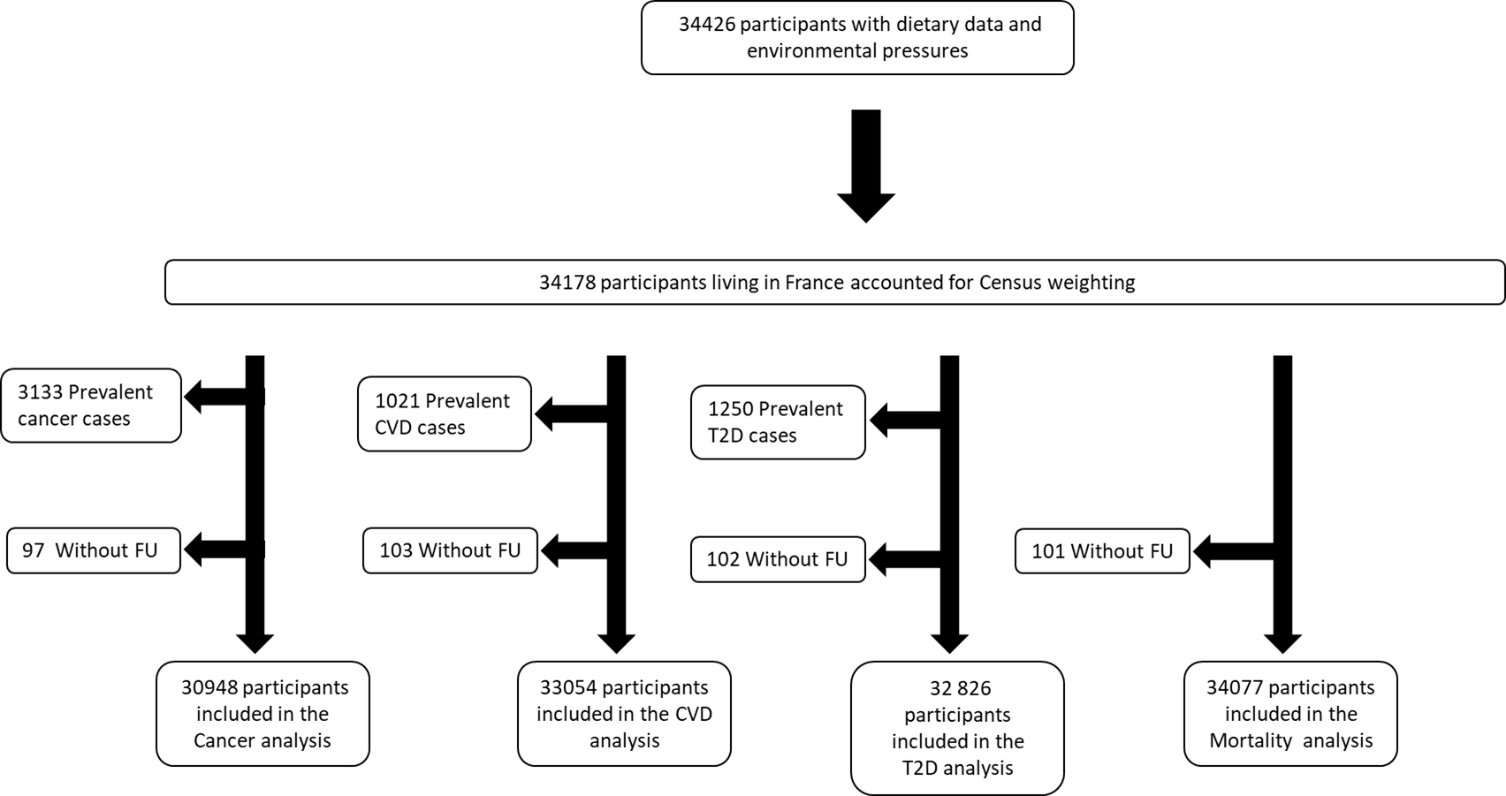


# Supplemental Figure 1: Flowchart, NutriNet-Santé cohort, France, 2014-2024 FU: Follow-up; CVD: cardiovascular diseases; T2D: Type 2 diabetes


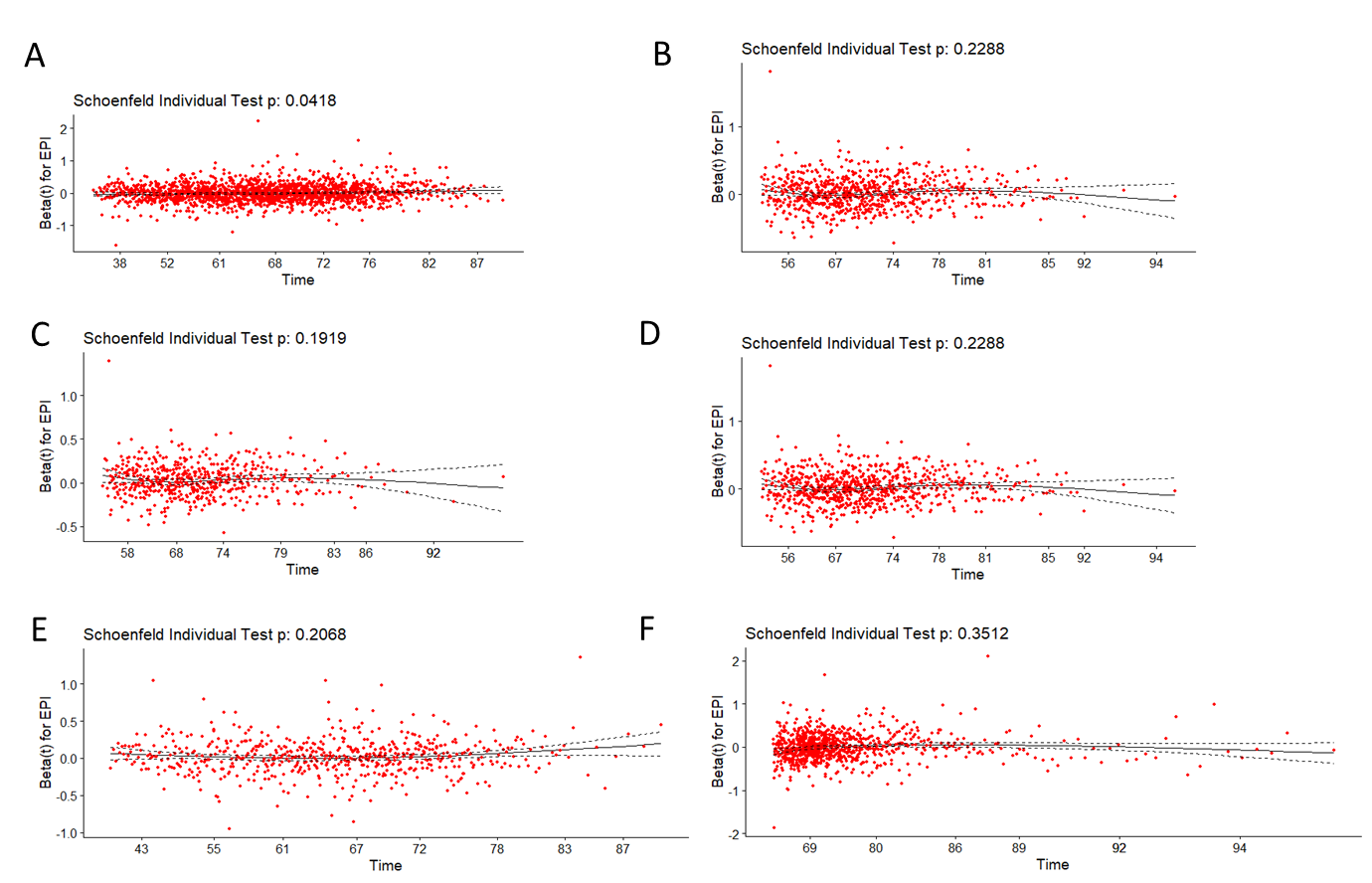


# Supplemental Figure 2: Correlations between Schoenfeld residuals and timescale (age, y) from multivariable Cox models between EPI and risk of chronic diseases and mortality, NutriNet-Santé study, 2014-2024, (n = 34,077).

Abbreviations: EPI, Environmental Pressure Index. Time is age and data are weighted

Panel A is cancer, B is cardiovascular diseases, C is coronary heart disease, D is Stroke, E is type 2 Diabetes, and F is mortality. Schoenfeld residuals plots according to time allow to check for the proportional hazard assumption. Multivariable Cox models are adjusted for age (time-scale), sex (male/female), physical activity level (low, moderate, high), smoking status (status as smoker, former smoker and non-smokers, and number of pack-year), energy intake (continuous, in kcal/d), number of 24-hour dietary records (continuous), educational attainment (<high-school degree, ≤3 years of higher education, >3 years of higher education), living status (cohabiting or not), occupational status (retired, unemployed, farmer/merchant/craftworker/company director, employee/manual worker, intermediate profession, managerial staff/intellectual profession, never employed), monthly income per unit consumption of the household (non-communicated, <1,200 €, 1,200 – 1,800 €, 1,800 – 3,700 €, ≥ 3,700 €), body mass index (BMI) (continuous, in kg/m²), and family history of cancer, diabetes or cardiovascular diseases depending on the analysis. For the cancer analysis, height (continuous, in m) and, for women, number of children, hormone replacement, age at menarche and contraceptive use were included in the model.


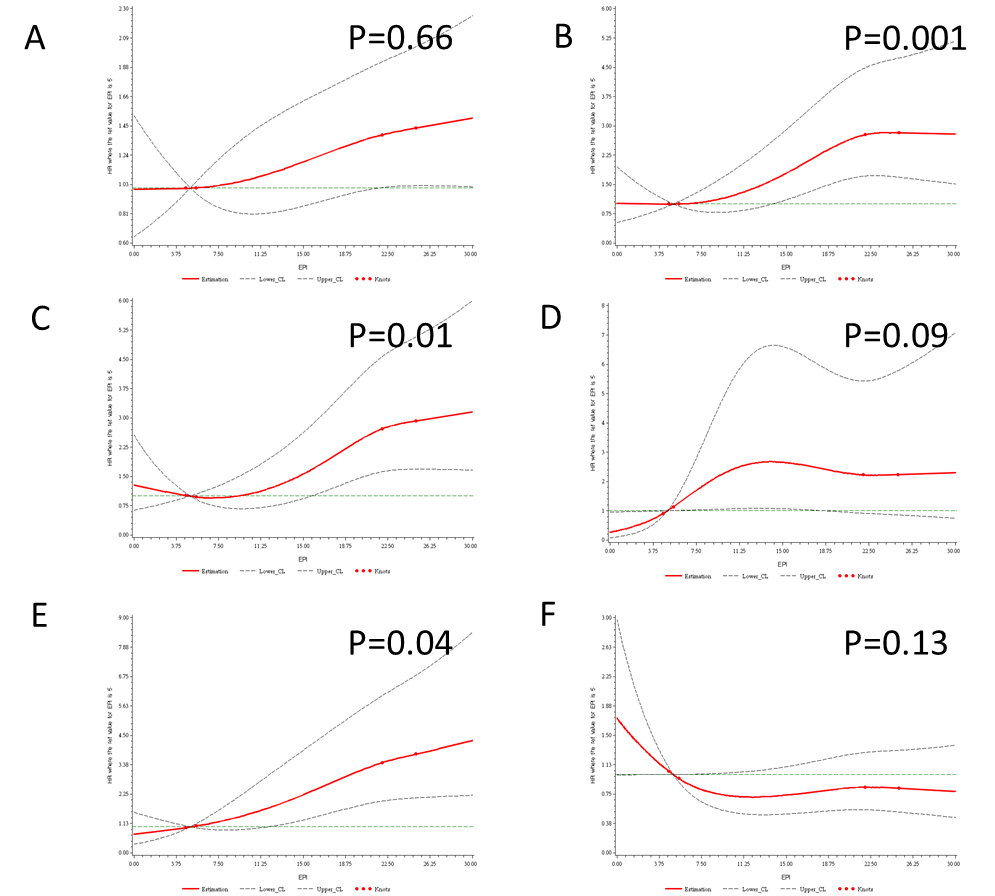


# Supplemental Figure 3: Restricted cubic spline plots of the association between EPI and risk of chronic diseases and mortality, NutriNet-Santé study, 2014–2024 (n = 34,077).

Panel A is cancer, B is cardiovascular diseases, C is coronary heart disease, D is Stroke, E is type 2 Diabetes, and F is mortality. Multivariable Cox models using Restricted Cubic Spline (RCS) SAS Macro®^37^ and adjusted for age (time-scale), sex (male/female), physical activity level (low, moderate, high), smoking status (status as smoker, former smoker and non-smokers, and number of pack-year), energy intake (continuous, in kcal/d), number of 24-hour dietary records (continuous), educational attainment (<high-school degree, ≤3 years of higher education, >3 years of higher education), living status (cohabiting or not), occupational status (retired, unemployed, farmer/merchant/craftworker/company director, employee/manual worker, intermediate profession, managerial staff/intellectual profession, never employed), monthly income per unit consumption of the household (non-communicated, <1,200 €, 1,200 – 1,800 €, 1,800 – 3,700 €, ≥ 3,700 €), body mass index (BMI) (continuous, in kg/m²), and family history of cancer, diabetes or cardiovascular diseases depending on the analysis. For the cancer analysis, height (continuous, in m) and, for women, number of children, hormone replacement, age at menarche, and contraceptive use were included in the model. P referred to the test for non-linearity.


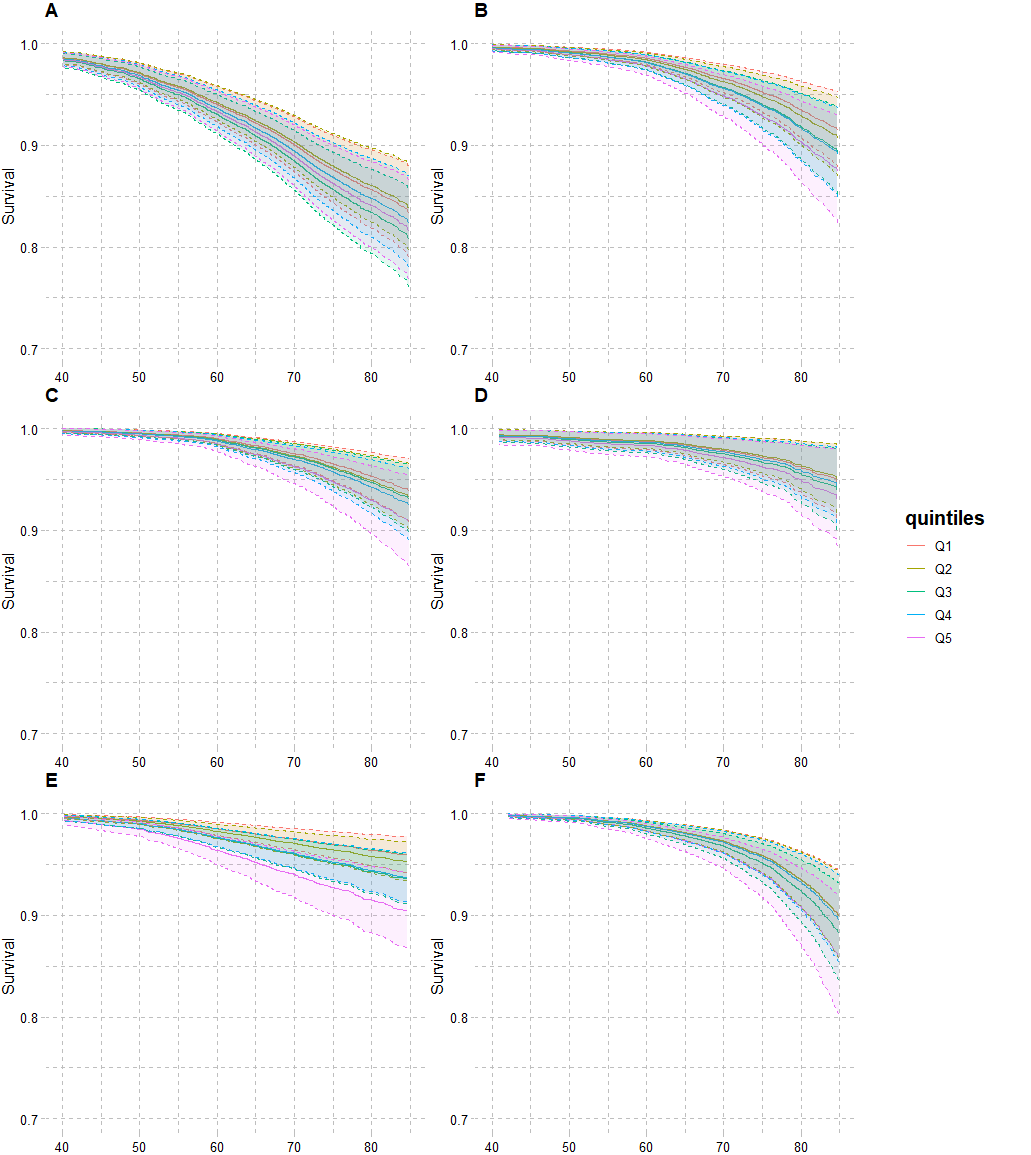


# Supplemental Figure 4: Standardized survival curves by quintiles of EPI for chronic diseases or mortality, NutriNet-Santé cohort, France, 2014-2024

Abbreviations; CHD, coronary heart diseases; CVD, cardiovascular diseases; T2D, type 2 diabetes.

The x-axis is age (in y). Each curve depicts the age-standardized probability of survival (marginal survival curves) for chronic disease (cancers, cardiovascular diseases, type 2 diabetes) and total mortality. Standardized survival is based on the counterfactual model for the covariates and is interpreted as a change in risk associated with the changes in diet for a fixed covariate profile. The covariate profile is for women, employees, without cancer family history, living in couple, physically active, education attainment ≤3y after high school, former smokers, income between 1200 and 1800€/month/unit consumption, with cumulative tobacco consumption of 5.5 pack year, 2000 Kcal/d, body mass index of 24 kg/m², and for risk of cancer height of 1.66m, no contraceptive and hormonal replacement use, age at menarche <12y, and 2 children.


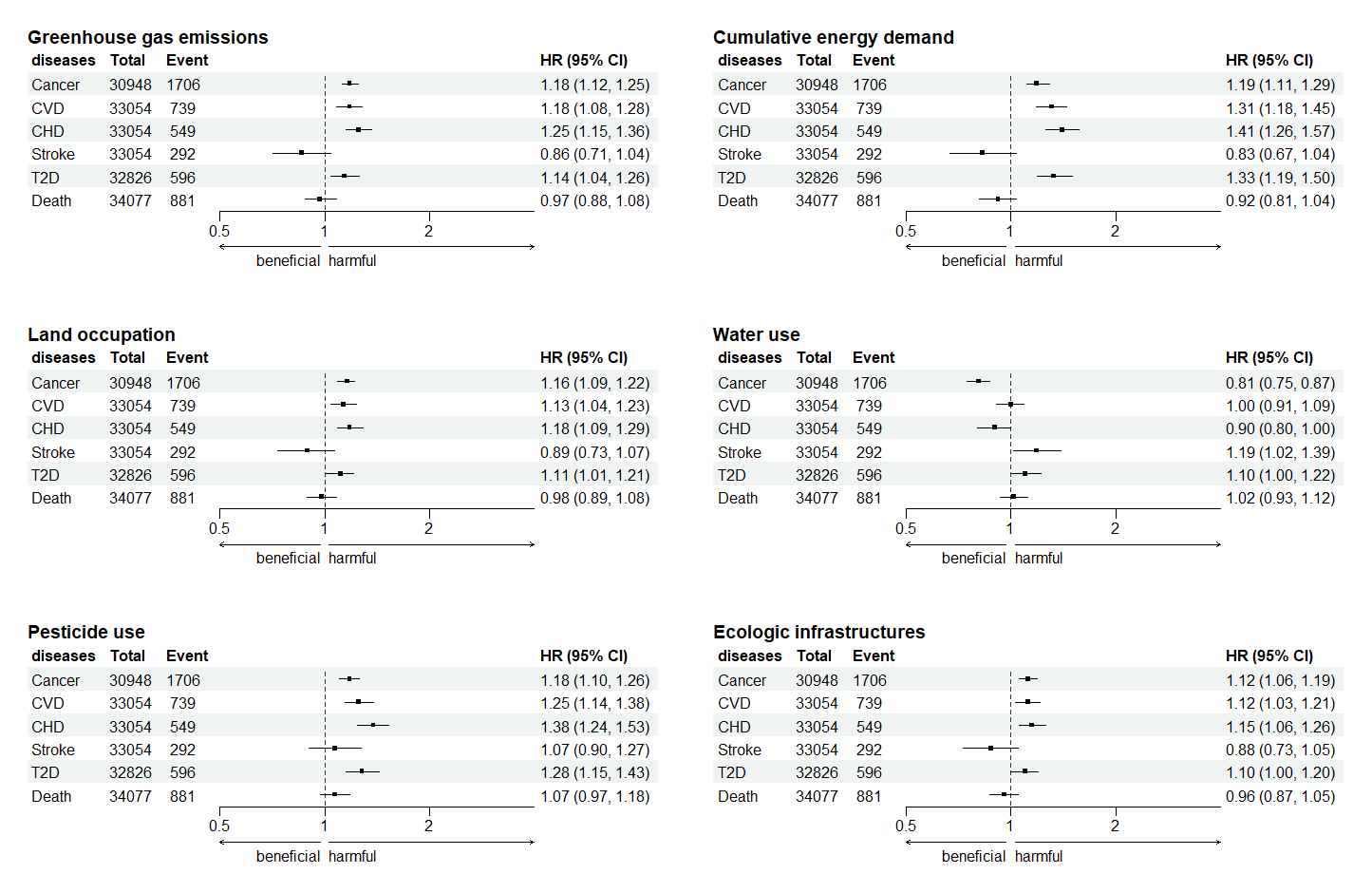


# Supplemental Figure 5: Association between each individual environmental indicator and chronic diseases and mortality (NutriNet-Santé, 2014-2024)

Abbreviations; CHD, coronary heart diseases; CVD, cardiovascular diseases; T2D, type 2 diabetes.

Values are number (total and cases of disease), HR (95% CI). HR (95% CI) are extracted from multivariable Cox models are adjusted for age (time-scale), sex (male/female), physical activity level (low, moderate, high), smoking status (status as smoker, former smoker and non-smokers, and number of pack-year), energy intake (continuous, in kcal/d), number of 24-hour dietary records (continuous), educational attainment (<high-school degree, ≤3 years of higher education, >3 years of higher education), living status (cohabiting or not), occupational status (retired, unemployed, farmer/merchant/craftworker/company director, employee/manual worker, intermediate profession, managerial staff/intellectual profession, never employed), monthly income per unit consumption of the household (non-communicated, <1,200 €, 1,200 – 1,800 €, 1,800 – 3,700 €, ≥ 3,700 €), body mass index (BMI) (continuous, in kg/m^²^), and family history of cancer, diabetes or cardiovascular diseases depending on the analysis. For the cancer analysis, height (continuous, in m) and, for women, number of children, hormone replacement, age at menarche, and contraceptive use were included in the model.

# Supplemental Table 1: Description of weight according to characteristics categories (NutriNet-Santé cohort, France, 2014 (n = 34,077)

|  | **N** | **Mean** | **SD** |
| --- | --- | --- | --- |
| Sex |  |  |  |
| Women | 25,723 | 0·69 | 2·39 |
| Men | 8,354 | 1·94 | 7·92 |
| Age (years) |  |  |  |
| <30 | 2,453 | 2·26 | 9·33 |
| 30-45 | 7,030 | 1·27 | 5·97 |
| 45-60 | 11,202 | 0·85 | 3·97 |
| >60 | 13,392 | 0·75 | 1·6 |
| Education |  |  |  |
| < High school diploma | 7,157 | 2·84 | 8·39 |
| High school | 5,020 | 1·05 | 4·24 |
| ≤3 years after high school | 10,458 | 0·39 | 1·94 |
| >3 years after high school | 11,442 | 0·39 | 1·12 |
| Smoking status |  |  |  |
| Never smoker | 16,610 | 0·97 | 4·26 |
| Former smoker | 13,754 | 0·99 | 4·38 |
| Smoker | 3,713 | 1·16 | 5·57 |
| Occupation |  |  |  |
| Unemployed | 14,06 | 1·03 | 5·64 |
| Retired | 12,375 | 0·76 | 1·61 |
| Employee/manual worker | 4,955 | 2·14 | 9 |
| Craftsman, trader, business manager, farmer | 631 | 2·41 | 7·81 |
| Intermediate occupation | 5,095 | 0·97 | 4·34 |
| Executive or higher intellectual profession | 7,186 | 0·43 | 1·45 |
| Never employed | 2,429 | 1·27 | 4·15 |
| Income |  |  |  |
| <1200€ | 2,387 | 2·02 | 7·62 |
| 1200–1800€ | 7,890 | 1·24 | 4·6 |
| 1800–3700€ | 9,313 | 0·89 | 3·9 |
| >3700€ | 10,787 | 0·47 | 1·61 |
| NA | 3,700 | 1·65 | 7·31 |
| Living status |  |  |  |
| Cohabiting | 28,626 | 0·95 | 4·24 |
| Alone | 5,451 | 1·24 | 5·53 |
| Physical Activity |  |  |  |
| Unknown | 3,688 | 1·32 | 6·11 |
| High | 11,318 | 1·02 | 4·47 |
| Medium | 12,498 | 0·83 | 3·48 |
| Low | 6,573 | 1·09 | 5 |

# Supplemental Table 2: Dietary Consumption according to EPI quintiles (NutriNet-Santé cohort, 2014, n=34,077)^1,2^

| ***Food consumption*** (g/d)^2^ | Q1 | Q2 | Q3 | Q4 | Q5 |
| --- | --- | --- | --- | --- | --- |
| Offal | 1·51 (2·97) | 2·41 (4·08) | 3·40 (5·41) | 5·29 (7·17) | 8·74 (42·34) |
| Animal fat | 24·15 (20·90) | 32·49 (28·95) | 36·82 (33·78) | 37·91 (28·47) | 95·02 (341·00) |
| Sweetened beverages | 3·26 (4·94) | 4·47 (6·39) | 5·51 (6·82) | 5·84 (6·59) | 7·75 (8·80) |
| Processed meat | 79·54 (153·66) | 82·83 (121·42) | 83·94 (157·54) | 117·71 (155·02) | 124·12 (193·14) |
| Wholegrain | 597·48 (410·54) | 648·80 (441·57) | 702·88 (498·37) | 799·64 (506·52) | 843·67 (586·88) |
| Refined cereals | 54·70 (141·64) | 40·81 (100·23) | 58·23 (129·29) | 69·30 (164·05) | 87·02 (194·25) |
| Fruits | 11·91 (12·19) | 17·44 (17·36) | 21·49 (18·09) | 28·26 (20·36) | 38·99 (36·41) |
| Fruit juice | 77·27 (102·97) | 61·50 (76·72) | 70·76 (106·86) | 59·09 (69·82) | 59·93 (81·93) |
| Milk | 105·40 (87·07) | 125·38 (92·65) | 142·35 (130·05) | 149·74 (92·76) | 168·39 (124·55) |
| Legumes | 171·30 (135·91) | 220·18 (185·57) | 254·27 (204·06) | 300·97 (222·27) | 460·59 (502·51) |
| Vegetable fat | 44·73 (66·72) | 69·93 (100·64) | 85·59 (109·99) | 93·85 (110·18) | 121·33 (160·12) |
| Nuts | 42·93 (109·74) | 60·38 (127·36) | 62·66 (136·67) | 73·98 (137·77) | 81·81 (170·00) |
| Fat and sweet products | 23·20 (50·62) | 24·97 (63·35) | 15·72 (29·37) | 17·86 (39·41) | 20·04 (32·27) |
| Fish | 20·98 (13·77) | 23·92 (17·52) | 27·12 (16·77) | 34·09 (21·18) | 39·49 (28·88) |
| Pork | 9·52 (18·06) | 9·10 (19·61) | 6·92 (15·17) | 7·15 (14·47) | 8·43 (19·04) |
| Potatoes | 62·65 (50·29) | 67·89 (52·08) | 73·79 (56·92) | 89·25 (58·30) | 112·87 (90·23) |
| Dairy products | 24·92 (30·69) | 31·21 (29·75) | 45·93 (45·42) | 48·84 (44·03) | 58·58 (64·39) |
| Ruminant meat | 5·18 (6·86) | 9·53 (10·61) | 12·90 (14·27) | 19·99 (16·34) | 38·44 (111·00) |
| Plant-based substitutes | 18·31 (17·55) | 23·13 (19·67) | 29·50 (36·09) | 32·38 (25·39) | 40·39 (55·34) |
| Vegetables | 116·59 (103·69) | 153·34 (115·31) | 198·32 (140·96) | 216·84 (145·19) | 261·12 (192·97) |
| Poultry | 14·54 (13·93) | 25·86 (19·89) | 37·90 (26·25) | 49·80 (27·45) | 87·78 (71·07) |
| Eggs | 56·71 (119·88) | 44·21 (138·52) | 27·40 (95·31) | 20·44 (70·31) | 29·28 (128·02) |

Abbreviations: EPI, environmental pressures index; Q, quintiles

^1^Values are unadjusted mean (SD), weighted on Census

^2^P-values for linear contrast across quintiles are <0·05

# Supplemental Table 3: Association between EPI and risk of chronic diseases and death, main analyses (NutriNet-Santé cohort, France, 2014–2024 (n = 34,077)^1^

|  | **Continuous variable^2^** | **P-value** |  | **Sex-specific** | **quintile** |  |  | **P-trend^3^** |
| --- | --- | --- | --- | --- | --- | --- | --- | --- |
|  |  |  | **Q1** | **Q2** | **Q3** | **Q4** | **Q5** |  |
| Cancer |  |  |  |  |  |  |  |  |
| n cases (unweighted) | 1,706 |  | 282 | 301 | 379 | 411 | 333 |  |
| person-year | 226017 |  | 41,939 | 44,040 | 42,964 | 44,192 | 44,547 |  |
| Model 1 (main) | 1·15 (1·03-1·28) | 0·01 |  | 0·71 (0·58-0·87) | 1·46 (1·22-1·75) | 0·97 (0·79-1·19) | 1·21 (0·95-1·55) | 0·02 |
| Cardiovascular diseases |  |  |  |  |  |  |  |  |
| n cases (unweighted) | 739 |  | 115 | 136 | 156 | 180 | 152 |  |
| person-year | 244,734 |  | 43,708 | 45,442 | 45,313 | 46,507 | 46,666 |  |
| Model 1 (main) | 1·40 (1·22-1·61) | <0·0001 |  | 1·29 (0·99-1·68) | 1·38 (1·05-1·81) | 1·67 (1·26-2·20) | 1·94 (1·38-2·72) | 0·0001 |
| Coronary heart diseases |  |  |  |  |  |  |  |  |
| n cases (unweighted) | 549 |  | 86 | 103 | 106 | 133 | 121 |  |
| person-year | 244,734 |  | 43,708 | 45,442 | 45,313 | 46,507 | 46,666 |  |
| Model 1 (main) | 1·50 (1·29-1·73) | <0·0001 |  | 1·25 (0·94-1·66) | 0·94 (0·69-1·29) | 1·55 (1·15-2·10) | 2·19 (1·53-3·14) | 0·0001 |
| Stroke |  |  |  |  |  |  |  |  |
| n cases (unweighted) | 292 |  | 54 | 53 | 65 | 64 | 56 |  |
| person-year | 244,734 |  | 43,708 | 45,442 | 45,313 | 46,507 | 46,666 |  |
| Model 1 (main) | 1·04 (0·80-1·36) | 0·76 |  | 1·75 (1·08-2·82) | 2·34 (1·45-3·77) | 1·37 (0·80-2·34) | 1·26 (0·65-2·41) | 0·76 |
| Type 2 diabetes |  |  |  |  |  |  |  |  |
| n cases (unweighted) | 596 | 71 | 90 | 125 | 141 | 169 | 596 |  |
| person-year | 244,248 |  | 42,709 | 46,162 | 44,326 | 46,054 | 46,554 |  |
| Model 1 (main) | 1·50 (1·29-1·74) | <0·0001 |  | 0·37 (0·26-0·52) | 0·95 (0·71-1·28) | 1·13 (0·85-1·52) | 1·41 (0·99-2·02) | 0·0001 |
| Death |  |  |  |  |  |  |  |  |
| n cases (unweighted) | 881 | 146 | 160 | 187 | 190 | 198 | 881 |  |
| person-year | 256,891 |  | 45,850 | 48,355 | 47,737 | 49,366 | 49,337 |  |
| Model 1 (main) | 1·01 (0·87-1·18) | 0·85 |  | 0·95 (0·74-1·21) | 1·20 (0·94-1·54) | 0·93 (0·71-1·21) | 0·95 (0·68-1·33) | 0·79 |

^1^HR (Hazard Ratio) and 95% CI (95% confidence interval) are derived from multivariable Cox proportional hazard, Q: Quintile.

^2^ by increment of 1SD

^3^ P-value of Wald test for quintile as an ordinal variable

^4^ The main model (M1) is a weighted multivariable Cox proportional hazard model adjusted for age (time-scale), sex (male/female), physical activity level (low, moderate, high), smoking status (status as current smoker, former smoker and non-smokers, and number of pack-year), energy intake (continuous, in kcal/d), number of 24-hour dietary records (continuous), educational attainment (<high-school degree, ≤3 years of higher education, >3 years of higher education), living status (cohabiting or not), occupational status (retired, unemployed, farmer/merchant/craftworker/company director, employee/manual worker, intermediate profession, managerial staff/intellectual profession, never employed), monthly income per unit consumption of the household (non-communicated, <1,200 €, 1,200 – 1,800 €, 1,800 – 3,700 €, ≥ 3,700 €), body mass index (BMI) (continuous, in kg/m²), and family history of cancer, diabetes or cardiovascular diseases depending on the analysis. For the cancer analysis, height (continuous, in m) and, for women, number of children, hormone replacement and contraceptive use were included in the model.

# Supplemental Table 4: Association between EPI and risk of chronic diseases and death, main and sensitivity analyses (NutriNet-Santé cohort, France, 2014–2024 (n = 34,077)^1^

|  | **Continuous variable^2^** | **P-value** |  |  |  |  |  | **P-trend^3^** |
| --- | --- | --- | --- | --- | --- | --- | --- | --- |
|  |  |  | **Q1** | **Q2** | **Q3** | **Q4** | **Q5** |  |
| ***Sensitive analysis 1^4^*** |  |  |  |  |  |  |  |  |
| Cancer | 1·11 (1·04-1·19) | 0·001 |  | 0·71 (0·58-0·87) | 1·46 (1·23-1·73) | 0·97 (0·81-1·16) | 1·21 (1·02-1·44) | 0·002 |
| Cardiovascular diseases | 1·05 (0·96-1·14) | 0·27 |  | 1·18 (0·91-1·52) | 1·16 (0·90-1·50) | 1·27 (0·99-1·62) | 1·18 (0·92-1·52) | 0·19 |
| Coronary heart diseases | 1·10 (1·01-1·21) | 0·04 |  | 1·13 (0·85-1·49) | 0·78 (0·58-1·06) | 1·14 (0·87-1·49) | 1·26 (0·97-1·65) | 0·06 |
| Stroke | 0·96 (0·82-1·13) | 0·62 |  | 1·72 (1·07-2·76) | 2·28 (1·46-3·58) | 1·32 (0·81-2·13) | 1·17 (0·71-1·93) | 0·68 |
| Type 2 diabetes | 1·14 (1·04-1·25) | 0·01 |  | 0·33 (0·24-0·47) | 0·82 (0·62-1·08) | 0·90 (0·70-1·16) | 0·93 (0·72-1·20) | 0·01 |
| Death | 0·95 (0·87-1·04) | 0·28 |  | 0·93 (0·74-1·18) | 1·17 (0·93-1·48) | 0·89 (0·70-1·12) | 0·88 (0·70-1·11) | 0·20 |
| ***Sensitive analysis 2^5^*** |  |  |  |  |  |  |  |  |
| Cancer | 1·15 (1·02-1·30) | 0·02 |  | 0·75 (0·60-0·94) | 1·60 (1·31-1·96) | 0·95 (0·75-1·20) | 1·23 (0·93-1·63) | 0·06 |
| Cardiovascular diseases | 1·41 (1·21-1·64) | <0.0001 |  | 1·29 (0·96-1·73) | 1·34 (0·99-1·82) | 1·74 (1·28-2·37) | 1·96 (1·34-2·87) | 0·0001 |
| Coronary heart diseases | 1·50 (1·28-1·76) | <0.0001 |  | 1·37 (0·99-1·88) | 0·90 (0·63-1·30) | 1·74 (1·24-2·44) | 2·57 (1·71-3·84) | 0·0001 |
| Stroke | 1·03 (0·77-1·37) | 0·86 |  | 1·60 (0·97-2·62) | 2·01 (1·22-3·31) | 1·19 (0·68-2·09) | 0·92 (0·45-1·85) | 0·67 |
| Type 2 diabetes | 1·59 (1·36-1·87) | <0.0001 |  | 0·34 (0·23-0·50) | 0·97 (0·71-1·32) | 1·14 (0·83-1·57) | 1·60 (1·09-2·35) | 0·0001 |
| Death | 1·03 (0·88-1·20) | 0·72 |  | 0·99 (0·77-1·28) | 1·30 (1·01-1·69) | 0·97 (0·73-1·29) | 0·89 (0·62-1·26) | 0·66 |
| ***Sensitive analysis 3^6^*** |  |  |  |  |  |  |  |  |
| Cancer | 1·08 (0·95-1·23) | 0·22 |  | 0·80 (0·64-1·00) | 1·01 (0·81-1·25) | 0·97 (0·77-1·23) | 1·08 (0·81-1·42) | 0·28 |
| Cardiovascular diseases | 1·35 (1·14-1·59) | 0·001 |  | 1·37 (1·00-1·87) | 1·45 (1·05-2·02) | 1·79 (1·27-2·52) | 2·07 (1·38-3·12) | 0·0003 |
| Coronary heart diseases | 1·42 (1·19-1·71) | 0·0001 |  | 1·23 (0·87-1·75) | 1·08 (0·74-1·59) | 1·54 (1·05-2·26) | 2·28 (1·46-3·57) | 0·001 |
| Stroke | 1·06 (0·78-1·44) | 0·69 |  | 1·47 (0·89-2·46) | 1·96 (1·17-3·27) | 1·73 (0·98-3·06) | 1·38 (0·68-2·82) | 0·22 |
| Type 2 diabetes | 1·39 (1·17-1·64) | 0·0001 |  | 0·56 (0·37-0·85) | 1·24 (0·86-1·78) | 1·06 (0·72-1·56) | 1·33 (0·85-2·07) | 0·02 |
| Death | 1·00 (0·85-1·18) | 0·96 |  | 0·78 (0·59-1·03) | 1·20 (0·91-1·59) | 0·94 (0·69-1·27) | 0·98 (0·68-1·41) | 0·69 |
| ***Sensitive analysis 4^7^*** |  |  |  |  |  |  |  |  |
| Cancer | 1·08 (0·98-1·20) | 0·13 |  | 0·87 (0·69-1·10) | 1·11 (0·89-1·38) | 0·79 (0·62-1·00) | 1·17 (0·95-1·46) | 0·23 |
| Cardiovascular diseases | 0·95 (0·84-1·08) | 0·45 |  | 1·44 (1·08-1·91) | 1·32 (0·99-1·76) | 1·24 (0·93-1·66) | 1·15 (0·86-1·54) | 0·99 |
| Coronary heart diseases | 1·01 (0·87-1·17) | 0·91 |  | 1·47 (1·05-2·05) | 1·22 (0·87-1·72) | 1·29 (0·92-1·80) | 1·27 (0·91-1·78) | 0·53 |
| Stroke | 0·79 (0·64-0·99) | 0·04 |  | 1·27 (0·82-1·96) | 1·28 (0·83-1·96) | 0·94 (0·59-1·48) | 0·85 (0·53-1·34) | 0·15 |
| Type 2 diabetes | 1·44 (1·27-1·63) | <0.0001 |  | 1·40 (1·00-1·95) | 1·44 (1·03-2·00) | 1·26 (0·90-1·77) | 2·40 (1·77-3·26) | 0·0001 |
| Death | 0·93 (0·83-1·05) | 0·254 |  | 0·88 (0·68-1·13) | 1·03 (0·81-1·31) | 0·97 (0·76-1·24) | 0·85 (0·66-1·09) | 0·41 |
| ***Sensitive analysis 5^8^*** |  |  |  |  |  |  |  |  |
| Cancer | 1·04 (0·94-1·15) | 0·43 |  | 1·00 (0·84-1·18) | 1·12 (0·94-1·32) | 1·08 (0·90-1·29) | 1·08 (0·87-1·34) | 0.31 |
| Cardiovascular diseases | 1·08 (0·93-1·26) | 0·30 |  | 1·03 (0·80-1·33) | 1·27 (0·98-1·64) | 1·18 (0·89-1·56) | 1·41 (1·01-1·96) | 0.03 |
| Coronary heart diseases | 1·13 (0·89-1·45) | 0·30 |  | 0·92 (0·62-1·36) | 1·17 (0·79-1·74) | 1·04 (0·67-1·61) | 1·33 (0·80-2·24) | 0.27 |
| Stroke | 1·07 (0·90-1·27) | 0·43 |  | 1·04 (0·78-1·39) | 1·18 (0·87-1·59) | 1·07 (0·77-1·48) | 1·42 (0·97-2·07) | 0.12 |
| Type 2 diabetes | 1·31 (1·14-1·51) | 0.0001 |  | 0·99 (0·72-1·36) | 1·51 (1·10-2·06) | 1·37 (0·98-1·90) | 1·97 (1·37-2·84) | 0.0001 |
| Death | 1·03 (0·90-1·18) | 0·65 |  | 0·90 (0·71-1·14) | 1·18 (0·93-1·48) | 0·99 (0·76-1·27) | 1·22 (0·91-1·64) | 0.15 |

^1^ HR (Hazard Ratio) and 95% CI (95% confidence interval) are derived from multivariable Cox proportional hazard, Q: Quintile.

^2^ by increment of 1SD

^3^ P-value of Wald test for quintile as an ordinal variable

^4^ Sensitivity analysis 1 is model M1 (see Footnote of the supplemental table 3) without adjustment for total energy intake (kcal/d)

^5^ Sensitivity analysis 2 is model M1 after removing early cases (in the first 1.5y of follow-up), 1,344 cancer, 609 cardiovascular diseases, 446 coronary heart diseases, 262 stroke, 506 type 2 diabetes, and 796 deaths

^6^ Sensitivity analysis 4 is a model weighed for census data after capping weight for census >95^th^ percentile at this value

^7^ Sensitivity analysis 3 is Marginal Structural Model additionally weighted for census data after removing participants with weight >10, 1,683 cancer, 729 cardiovascular diseases, 541 coronary heart diseases, 290 stroke, 591 type 2 diabetes, and 970 death

^8^ Sensitivity analysis 5 is a model without weighing for census data

# References

1 High Council of Public Health. Statement related to the revision of the 2017-2021 French Nutrition and Health Programme’s dietary guidelines for adults. Paris: Haut Conseil de la Santé Publique, 2017 https://www.hcsp.fr/explore.cgi/avisrapportsdomaine?clefr=653 (accessed Feb 5, 2019).

2 French Agency for Food, Environmental and Occupational Health Safety (Anses). Actualisation des repères du PNNS : élaboration des références nutritionnelles. Maison Alfort: ANSES, 2016 Available from: https://www.anses.fr/fr/system/files/NUT2012SA0103Ra-2.pdf.

3 Chaltiel D, Adjibade M, Deschamps V, *et al.* Programme National Nutrition Santé – guidelines score 2 (PNNS-GS2): development and validation of a diet quality score reflecting the 2017 French dietary guidelines – CORRIGENDUM. *Br J Nutr* 2021; **125**: 118–20.

4 Bui LP, Pham TT, Wang F, *et al.* Planetary Health Diet Index and risk of total and cause-specific mortality in three prospective cohorts. *Am J Clin Nutr* 2024; **120**: 80–91.

5 Willett W. Food in the Anthropocene: the EAT–Lancet Commission on healthy diets from sustainable food systems. *The Lancet* 2019; **393**: 447–92.

6 Council Regulation (EC) No 2018/848 of 30 may 2018 on on organic production and labelling of organic products and repealing Council Regulation (EC) No 834/2007. https://eur-lex.europa.eu/legal-content/EN/TXT/?uri=CELEX%3A02018R0848-20201114 (accessed April 24, 2021).

7 FAOSTAT. Detailed trade matrix. 2024. https://www.fao.org/faostat/en/#data/TM (accessed March 7, 2025).

8 Couturier C, Charru M, Doublet S, Pointereau P. Afterres2050_version2016. 75, voie du TOEC - CS 27608 31076 Toulouse Cedex 3, 2016 https://afterres2050.solagro.org/wp-content/uploads/2015/11/solagro_afterres2050_version2016.pdf (accessed Nov 25, 2024).

9 FAOSTAT. Food Balances. 2024. https://www.fao.org/faostat/en/#data/FBS (accessed March 7, 2025).

10 FAOSTAT. Data. 2024. https://www.fao.org/faostat/en/#data (accessed March 7, 2025).

11 Pointereau P, Langevin B, Gimaret M. DIALECTE, a comprehensive and quick tool to assess the agro-environmental performance of farms. 2012. http://ifsa.boku.ac.at/cms/index.php?id=ifsa2012.

12 Colomb V, Amar SA, Mens CB, *et al.* AGRIBALYSE®, the French LCI Database for agricultural products: high quality data for producers and environmental labelling. *OCL* 2015; **22**: D104.

13 Gac A, Tailleur A, Dauguet S. Allocation des impacts environnementaux à un produit ou à une activité agricole. 2020 https://www.arvalis.fr/sites/default/files/imported_files/___2-152978628971939315.pdf (accessed March 7, 2025).

14 PAN Europe, Pesticides Action Network Europe. Danish Pesticide Use Reduction Programme - to Benefit the Environment and the Health. 2005.

15 Ministère de l’Agriculture et de l’Alimentation. Indicateur de fréquence de traitements phytopharmaceutiques (IFT), Guide méthodologique Version 3 Avril 2018. Paris, France, 2018 https://agriculture.gouv.fr/indicateur-de-frequence-de-traitements-phytosanitaires-ift.

16 Gomiero T, Pimentel D, Paoletti MG. Environmental impact of different agricultural management practices: conventional vs. organic agriculture. *Crit Rev Plant Sci* 2011; **30**: 95–124.

17 Geiger F, Bengtsson J, Berendse F, *et al.* Persistent negative effects of pesticides on biodiversity and biological control potential on European farmland. *Basic and Applied Ecology* 2010; **11**: 97–105.

18 Pfister S, Koehler A, Hellweg S. Assessing the Environmental Impacts of Freshwater Consumption in LCA. *Environ Sci Technol* 2009; **43**: 4098–104.

19 Boulay A-M, Bare J, Benini L, *et al.* The WULCA consensus characterization model for water scarcity footprints: assessing impacts of water consumption based on available water remaining (AWARE). *Int J Life Cycle Assess* 2018; **23**: 368–78.

20 Hoekstra AY, Chapagain A, Aldaya MM, Mekonnen MM. Water Footprint Manual: State of the Art 2009, Water Footprint Network. Enschede, the Netherlands, 2009 www.waterfootprint.org/downloads/WaterFootprintManual2009.pdf.

21 Pfister S, Koehler A, Hellweg S. Assessing the Environmental Impacts of Freshwater Consumption in LCA. *Environ Sci Technol* 2009; **43**: 4098–104.

22 Hoekstra AY, editor. The water footprint assessment manual: setting the global standard. London ; Washington, DC: Earthscan, 2011.

23 WULCA. AWARE (Available WAter REmaining) Mission and Goals. WULCA. 2021. https://wulca-waterlca.org/aware/ (accessed March 7, 2025).

24 Banque nationale des prélèvements quantitatifs en eau. Eaufrance | Le service public d’information sur l’eau. Eaufrance. https://www.eaufrance.fr/ (accessed March 3, 202AD).

25 Agreste, la statistique agricole. Recensement agricole 2020 - Surface moyenne des exploitations agricoles en 2020 : 69 hectares en France métropolitaine et 5 hectares dans les DOM|Agreste, la statistique agricole. https://agreste.agriculture.gouv.fr/agreste-web/disaron/Pri2213/detail/ (accessed March 3, 2025).

26 Nair S, Johnson J, Wang C. Efficiency of Irrigation Water Use: A Review from the Perspectives of Multiple Disciplines. *Agronomy Journal* 2013; **105**: 351–63.

27 Agreste, la statistique agricole. Enquête pratiques culturales en grandes cultures et prairies 2017 - Principaux résultats (Version modifiée)|. 2020 https://agreste.agriculture.gouv.fr/agreste-web/disaron/Chd2009/detail/ (accessed March 3, 2025).

28 Huijbregts M. ReCiPe 2016 - A harmonized life cycle impact assessment method at midpoint and endpoint level Report I: Characterization. 2016.

29 Telepac. Formulaires et notices 2022- Déclaration des surfaces d’intérêt écologique (SIE) » (Métropole). https://www.telepac.agriculture.gouv.fr/telepac/html/public/aide/formulaires-2022.html (accessed March 3, 2024).

30 Solagro. Carte ‘pesticides’ - Adonis - Solagro. https://solagro.org/nos-domaines-d-intervention/agroecologie/carte-pesticides-adonis (accessed March 3, 2025).

31 Prévost M-C, Ménard J-L, Leclerc M-C. La maîtrise de la consommation d’eau en élevage bovin laitier. 2012; published online Dec. https://www.rmtelevagesenvironnement.org/backoffice/uploads/46_outil_maitrise_conso_eau_elevage_bovin_laitier.pdf (accessed March 7, 2025).

32 Consommations d’eau en élevage : entre sobriété et résilience. Institut de l’Élevage. 2022; published online Aug 12. https://idele.fr/detail-article/consommations-deau-en-elevage-entre-sobriete-et-resilience (accessed March 3, 2025).

33 Chesnaye NC, Stel VS, Tripepi G, *et al.* An introduction to inverse probability of treatment weighting in observational research. *Clinical Kidney Journal* 2022; **15**: 14–20.

34 Hernan MA, Robins JM. Estimating causal effects from epidemiological data. *JEpidemiolCommunity Health* 2006; **60**: 578–86.

35 Robins JM, Hernan J. Causal Inference: What If (the book), Chapman&Hall/CRC. Boca Raton, 2020 https://www.hsph.harvard.edu/miguel-hernan/causal-inference-book/.

36 Naimi AI, Moodie EEM, Auger N, Kaufman JS. Constructing Inverse Probability Weights for Continuous Exposures: A Comparison of Methods. *Epidemiology* 2014; **25**: 292–9.

37 Desquilbet L, Mariotti F. Dose-response analyses using restricted cubic spline functions in public health research. *Stat Med* 2010; **29**: 1037–57.
